# Supplementary material for: Theta-phase locking of single neurons during human spatial memory
Source: Nat Commun. 2025 Aug 11;16:7402. doi: 10.1038/s41467-025-62553-9 (PMC12339722; doi:10.1038/s41467-025-62553-9)
Supplement: Supplementary file 1 — Supplementary Information [file 41467_2025_62553_MOESM1_ESM.pdf]

Supplementary Information: Theta-phase locking of single neurons during human spatial memory

Tim A. Guth,[1,2]* Armin Brandt,[2] Peter C. Reinacher,[3,4] Andreas Schulze-Bonhage,[2] Joshua Jacobs,[5,6] Lukas Kunz[1]*

[1]Department of Epileptology, University Hospital Bonn, Bonn, Germany.

[2]Epilepsy Center, Medical Center – University of Freiburg, Faculty of Medicine, University of Freiburg, Freiburg, Germany.

[3]Department of Stereotactic and Functional Neurosurgery, Medical Center – University of Freiburg, Faculty of Medicine, University of Freiburg, Freiburg, Germany.

[4]Fraunhofer Institute for Laser Technology, Aachen, Germany.

[5]Department of Biomedical Engineering, Columbia University, New York, NY, USA.

[6]Department of Neurological Surgery, Columbia University Medical Center, New York, NY, USA.

*Correspondence: Tim.Guth@ukbonn.de; Lukas.Kunz@ukbonn.de.

Table S1. Participant information.

Participant index	Session index	Number of trials	Number of microwires	Number of units
1	1	21	32	19
1	2	39	32	18
2	1	32	64	64
2	2	32	64	63
3	1	40	32	50
3	2	28	32	55
4	1	40	24	28
4	2	40	24	12
5	1	40	48	48
5	2	40	48	40
6	1	40	48	33
6	2	40	48	23
7	1	24	48	52
8	1	32	48	42
8	2	24	48	36
9	1	40	48	13
10	1	40	48	42
10	2	40	48	37
11	1	40	48	48
12	1	40	48	56
13	1	40	16	19
14	1	40	48	48
15	1	40	32	24
16	1	35	32	45
17	1	40	48	62
18	1	40	32	28
18	2	40	32	20

Table S2. Fixed-effects estimates for the effect of brain region on pairwise phase consistency in a linear mixed-effects model.

Predictor	Degrees of freedom	*t*-value	*P*-value
**AMY versus EC**	1894	-4.236	<0.001
**AMY versus HC**	1894	-0.467	1
**AMY versus PHC**	1894	-8.446	<0.001
**AMY versus TP**	1894	-3.529	0.004
**EC versus HC**	1894	3.267	0.011
**EC versus PHC**	1894	-4.311	<0.001
**EC versus TP**	1894	0.347	1
**HC versus PHC**	1894	-7.317	<0.001
**HC versus TP**	1894	-2.649	0.081
**PHC versus TP**	1894	4.315	<0.001

Model formula: PPC ~ 1 + regions + (1|sessions) + (1|periods). ANOVA for linear mixed-effects model: $F(4) = 21.347$, $P < 0.001$. Analyses were performed with 210 neurons from the amygdala (AMY), 130 from the entorhinal cortex (EC), 125 from the hippocampus (HC), 76 from the parahippocampal cortex (PHC), and 92 from the temporal pole (TP). Note that the effect was driven by increased pairwise phase consistency in the parahippocampal cortex. *P*-values of pairwise comparisons are Bonferroni corrected for ten comparisons.

Table S3. Fixed-effects estimates for the effect of brain region on mean log-transformed power assigned to spikes in a linear mixed-effects model.

Predictor	Degrees of freedom	*t*-value	*P*-value
**AMY versus EC**	1894	-13.738	<0.001
**AMY versus HC**	1894	-26.237	<0.001
**AMY versus PHC**	1894	-9.253	<0.001
**AMY versus TP**	1894	-17.29	<0.001
**EC versus HC**	1894	-11.014	<0.001
**EC versus PHC**	1894	2.572	0.102
**EC versus TP**	1894	-3.789	0.002
**HC versus PHC**	1894	12.773	<0.001
**HC versus TP**	1894	6.32	<0.001
**PHC versus TP**	1894	-5.938	<0.001

Model formula: power ~ 1 + regions + (1|sessions) + (1|periods). ANOVA for linear mixed-effects model: $F(4) = 204.063$, $P < 0.001$. Analyses were performed with 210 neurons from the amygdala (AMY), 130 from the entorhinal cortex (EC), 125 from the hippocampus (HC), 76 from the parahippocampal cortex (PHC), and 92 from the temporal pole (TP). Note that the effects were driven by increased theta power in the hippocampus. *P*-values of pairwise comparisons are Bonferroni corrected for ten comparisons.

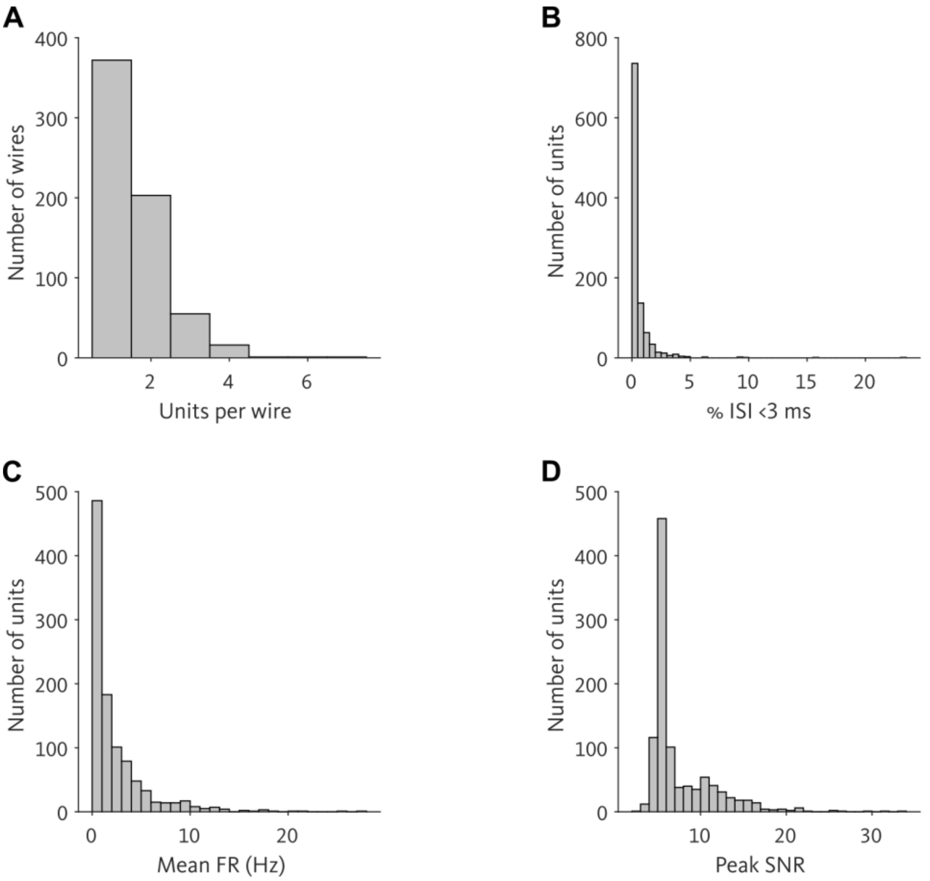

Figure S1. Quality assessment of single-neuron recordings. (**A**) Histogram of units per wire (*n* = 1025 units, *n* = 649 wires). On average, 1.58 ± 0.03 (mean ± SEM) units per wire were recorded. (**B**) Histogram of the percentages of inter-spike intervals that were shorter than 3 ms. On average, units exhibited 0.54 ± 0.04% (mean ± SEM) inter-spike intervals that were shorter than 3 ms. There were 7 units with values >5%. (**C**) Histogram of mean firing rates. On average, units exhibited mean firing rates of 2.31 ± 0.10 Hz (mean ± SEM). (**D**) Histogram of the mean waveform peak signal-to-noise ratio of each unit. On average, the signal-to-noise ratio of the mean waveform peak was 7.56 ± 0.12 (mean ± SEM). Source data are provided as a Source Data file.

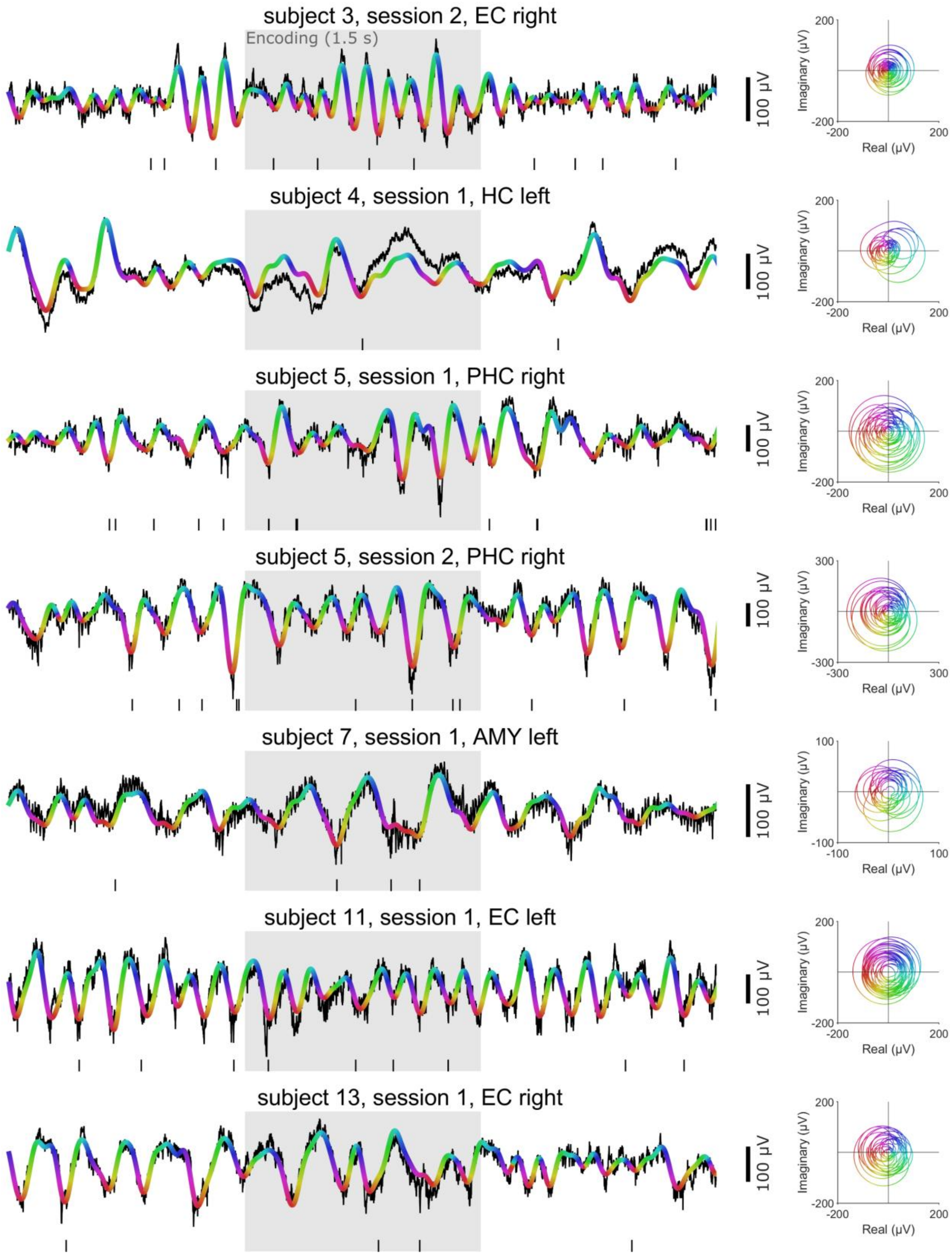

Figure S2. Examples of neuronal theta-phase locking in the human medial temporal lobe. Left: Examples of raw (black) and filtered (1–10 Hz; colored) local field potentials from different experimental sessions. Spike trains are shown as vertical lines below the local field potentials. Color indicates the instantaneous theta-phase angle estimated using a generalized phase approach (as in Fig. 2B), and gray areas indicate the 1.5-s encoding period. Right: Complex plane representation of the analytic signal, suggesting no relevant phase biases due to potential low-frequency intrusions. AMY, amygdala; EC, entorhinal cortex; HC, hippocampus; PHC, parahippocampal cortex. Source data are provided as a Source Data file.

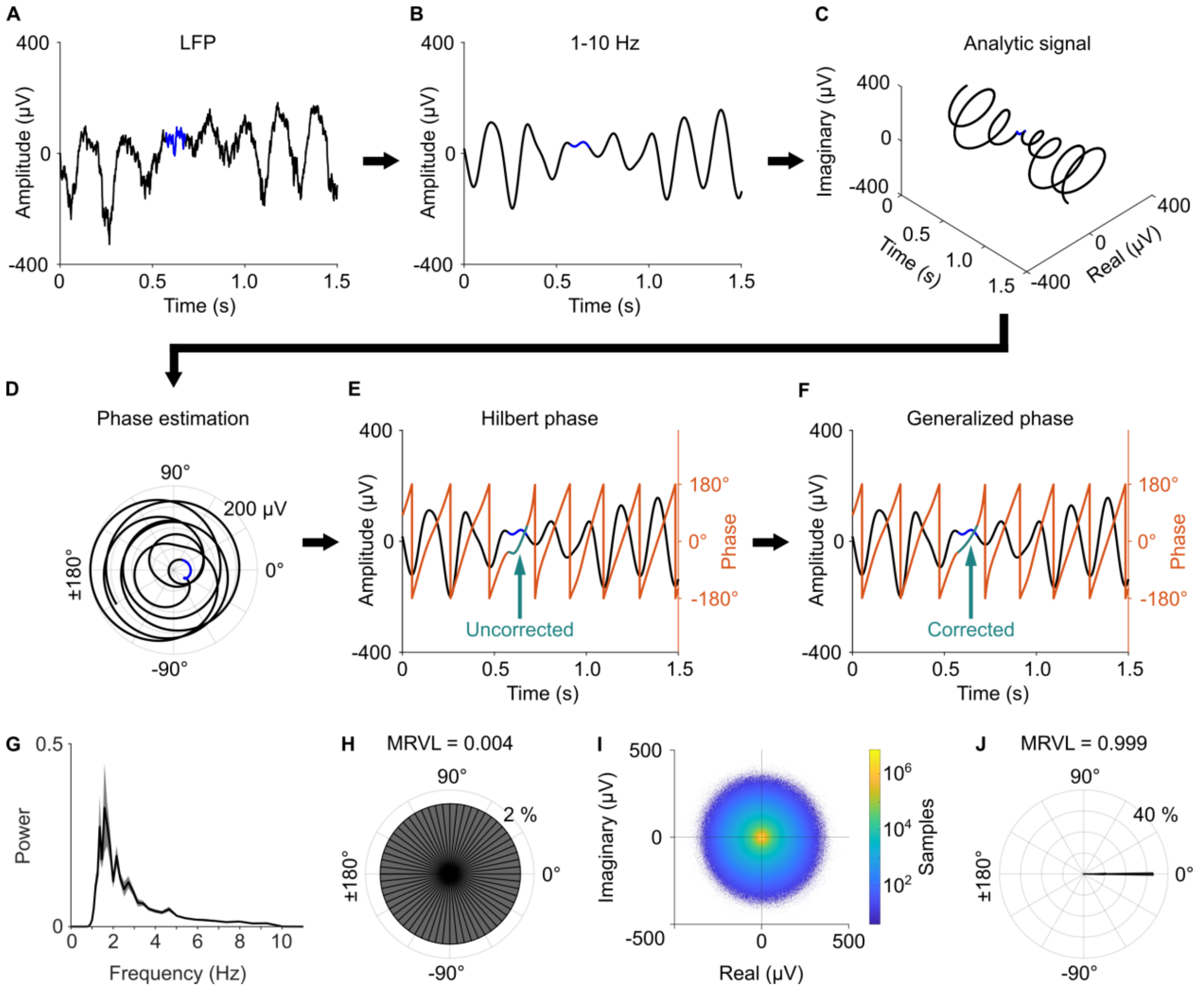

Figure S3. Generalized phase approach including illustration and control analyses. (**A–F**) Illustration of the generalized phase approach[1]. (**A**) Raw local field potential (LFP). (**B**) The LFP is filtered between 1–10 Hz. This removes low frequency content <1 Hz, which would shift the analytic signal representation in the complex plane. Low-frequency intrusions, such as DC shifts, can distort phase estimates, if not removed. (**C**) The filtered signal is Hilbert-transformed to obtain an analytic signal with a real and an imaginary part. (**D**) Phases are computed in the complex plane representation using the four-quadrant arctangent function. (**E**) Phase estimates can be distorted by high-frequency intrusions (turquoise arrow). (**F**) The generalized phase approach corrects for these distortions by identifying negative frequency epochs, in which estimated phase progression reverses direction or phase progresses with a frequency <1 Hz, and by replacing the phase values during these epochs with values from interpolation. (**G–J**) Control analyses showing that the resulting phase estimates are valid. (**G**) To examine whether our band-pass filter effectively removed activity below 1 Hz, we computed the grand-average power spectrum across all wires, showing negligible power beyond the 1–10 Hz frequency range. Black line, mean across microwires; gray shaded area, standard error of the mean across microwires. (**H**) To rule out a general bias in our phase estimates, we examined whether the instantaneous phases of all samples, pooled across all 502 wires, were distributed uniformly. A mean resultant vector length close to zero confirmed a practically uniform distribution of phase estimates. (**I**) 2D histogram of all analytic signals in the complex plane, showing a good centering of our signals in the complex plane. Accordingly, the means of the imaginary and real parts of the signals were close to zero (mean ± SEM: -0.034 ± 0.001 and -0.035i ± 0.001i, respectively). (**J**) Sample-wise phase difference between original phase estimates and phase estimates after local recentering in the complex plane using 10-second sliding-time windows. We performed this recentering to attenuate the effect of potential shifts in the complex plane due to potential low-frequency intrusions. The clustering of the phase differences around 0° indicates a negligible effect of such potential shifts on phase estimates. Source data are provided as a Source Data file.

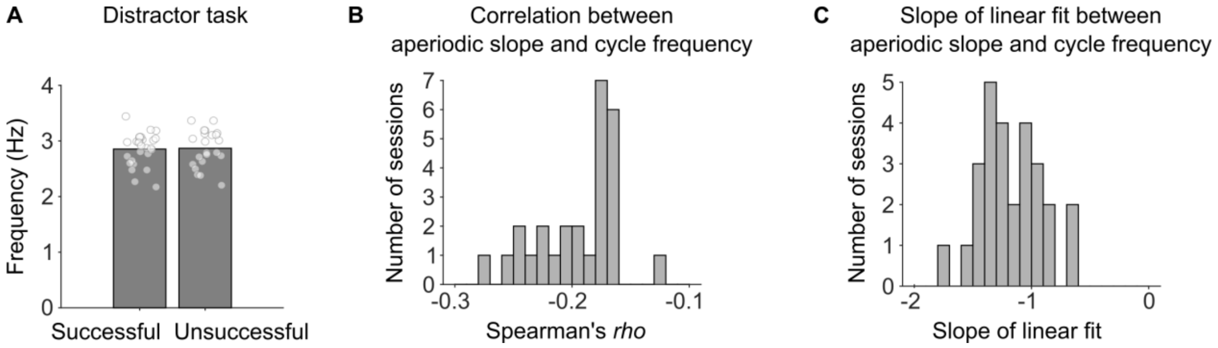

Figure S4. Theta frequencies as a function of distractor task performance and aperiodic slope. (**A**) As we included the 1–3 Hz range in our 1–10 Hz theta-frequency band, we performed a control analysis to test whether 1–3 Hz components of this band might be related to arousal as reflected by task disengagement. Hence, assuming that unsuccessful performance in the distractor task could reflect such a disengagement, we compared the median cycle-by-cycle frequency during successful versus unsuccessful distractor trials. We did not find a difference in median frequency between successful and unsuccessful trials (two-sided *t*-test: $t(26) = -0.173$, $P = 0.864$; $n = 27$ sessions), suggesting that 1–3 Hz frequencies did not increase due to task disengagement (i.e., lower arousal states). Bars show means of session-wise median frequencies; dots represent individual session medians. (**B**) Histogram showing mean Spearman's *rho*-values for the session-wise correlations between aperiodic slopes and cycle-by-cycle theta frequencies. Negative *rho*-values indicate that steeper slopes were associated with lower frequencies within the 1–10 Hz theta frequency range (two-sided *t*-test of correlation values versus 0: $t(26) = -29.200$, $P < 0.001$; $n = 27$ sessions). (**C**). To quantify aperiodic slope-associated frequency shifts, we calculated the session-wise mean slopes of linear fits between aperiodic slopes and cycle frequencies. A mean of approximately -1 indicates that an increase in aperiodic slope steepness by 1 corresponds to a frequency decrease of approximately 1 Hz. Source data are provided as a Source Data file.

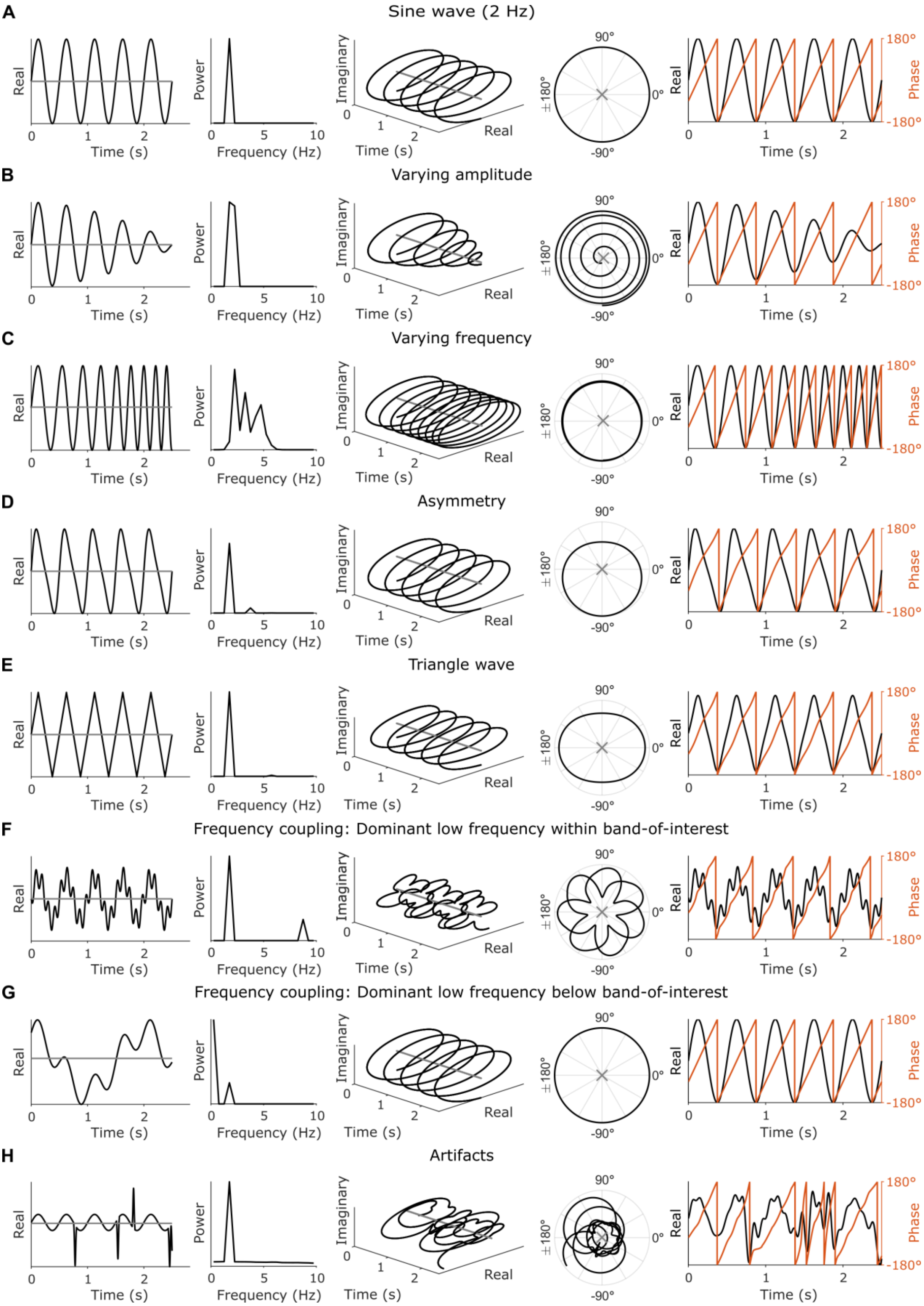

Figure S5. Generalized phase approach applied to simulated signals. (**A–H**) We tested the generalized phase approach using various simulated local field potentials (left). We plotted a power spectrum for each signal to inspect its different frequency components (middle left). Using the generalized phase approach including 1–10 Hz band-pass filtering, we obtained the analytic signal (middle) and instantaneous phases in the complex plane representation (middle right). We then inspected

the accuracy of the phase estimates (right). (**A**) Sine wave at 2 Hz. As expected, the generalized phase approach produced accurate phase estimates of this sinusoidal oscillation. (**B**) The phase estimates remained accurate despite varying amplitudes of the 2Hz sine wave. (**C**) Oscillation with varying frequency. The accurate phase estimates show that the generalized phase approach has a particular advantage over methods with narrow-band filtering for oscillations with variable frequencies. (**D–E**) It also provides accurate phase estimates for oscillations with their peak tilted to one side and for oscillations with a triangle-like shape. (**F**) Summation of a dominant low-frequency component within the frequency-band-of-interest (2 Hz) and a weaker high-frequency component (9 Hz). The generalized phase approach provides valid phase estimates for the low-frequency component at 2 Hz. This is because the generalized phase approach has a general preference for lower frequencies, caused by the 1/f-like shape of the power spectrum. (**G**) Summation of dominant low-frequency intrusions below the frequency-band-of-interest (0.5 Hz) and a weaker low-frequency component within the frequency-band-of-interest (2 Hz). The 1–10 Hz band-pass filter effectively removes the contribution of low-frequency intrusions, leading to valid phase estimates of the 2-Hz oscillation. (**H**) Artifacts can strongly distort phase estimates. For this reason, we excluded time periods during artifacts from further analysis.

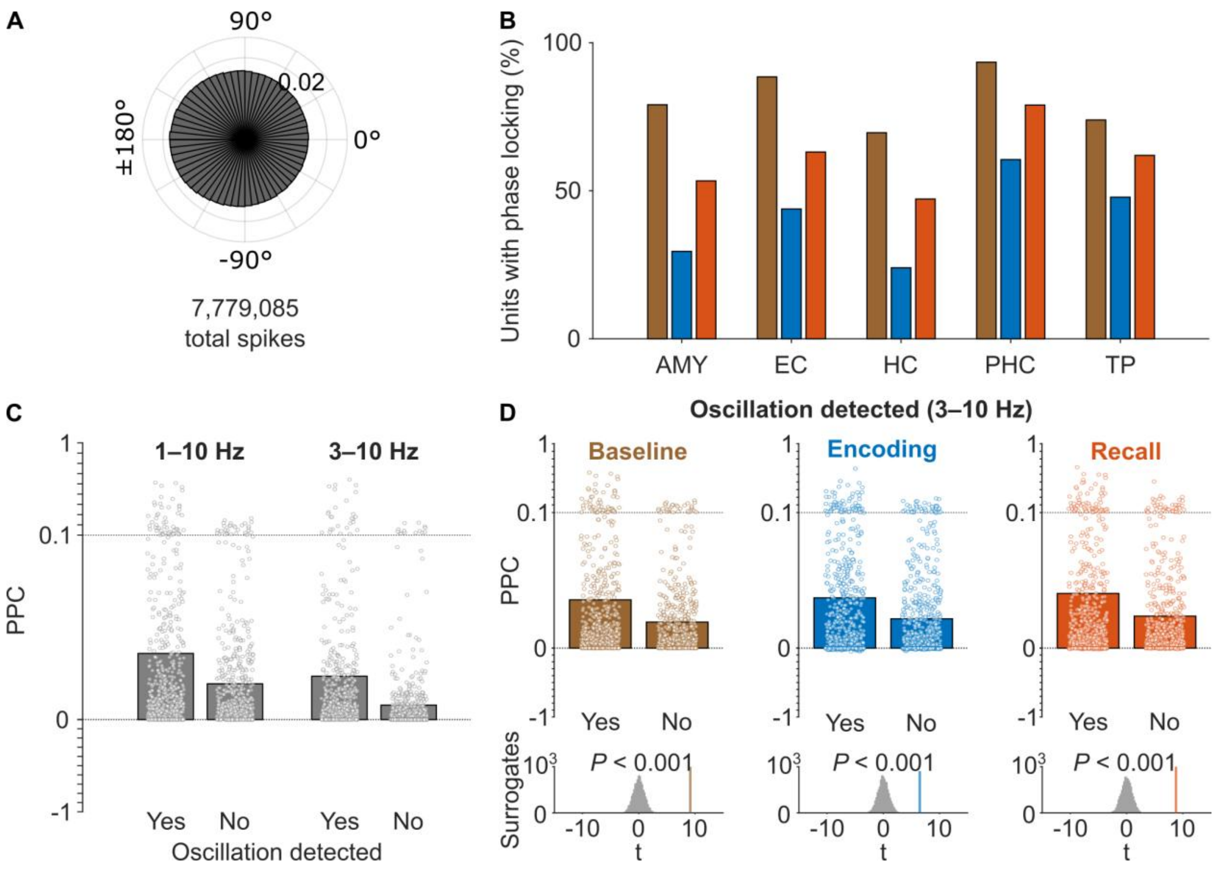

Figure S6. Theta-phase locking in the 3–10 Hz frequency range. We repeated some of our main analyses on theta-phase locking with the only difference of using a bandpass filter of 3–10 Hz before estimating generalized phase to enhance comparability with other studies on theta oscillation in humans that did not include low frequencies between 1–3 Hz. (**A**) General theta-phase locking across the entire experiment. Spikes pooled across all neurons preferentially occurred at the theta trough. This pattern was consistent with the analysis of theta-phase locking relative to the 1–10 Hz frequency range (Fig. 2C). (**B**) Percentages of units with significant phase locking. Percentages were similar but lower compared to the 1–10 Hz frequency range (Fig. 3A). (**C**) Theta-phase locking during the presence versus absence of clear theta oscillations analyzed within the 1–10 Hz and 3–10 Hz frequency bands. Bars show mean PPC values; dots represent PPC values of individual neurons. Y-axis is expanded between 0 and 0.1 to highlight the range containing most data points. We performed an ANOVA for a linear mixed-effects model with "frequency band" and "oscillation detected" as fixed effects and "session" as a random effect [PPC ~ 1 + frequency band * oscillation detected + (1 | session)]. There was a significant main effect of "frequency band" ($F(2660) = 15.942$, $P < 0.001$; $n = 666$ neurons) and a significant main effect of "oscillation detected" ($F(2660) = 32.198$, $P < 0.001$; $n = 666$ neurons). The interaction between "frequency band" and "oscillation detected" was not significant ($F(2660) = 0.036$, $P = 0.850$; $n = 666$ neurons). PPC values in the condition "1–10 Hz, no oscillation detected" were significantly lower than in the condition "3–10 Hz, oscillation detected" (two-sided t-test: $t(665) = -2.389$, $P = 0.017$; $n = 666$ neurons). These findings may help guide researchers in selecting a particular frequency-band-of-interest and in deciding whether to focus on signal segments with clear oscillations when investigating theta-phase locking. (**D**) 3–10 Hz theta-phase locking during the presence versus absence of clear theta oscillations detected by Bycycle, analyzed separately for baseline (left), encoding (middle), and recall (right). Bars show mean PPC values; dots represent PPC values of individual neurons. Y-axis is expanded between 0 and 0.1 to highlight the range containing most data points. Empirical t-values of paired t-tests were ranked in surrogate distributions of t-values to assess significance (plots at the bottom). P-values were Bonferroni corrected for three tests. Effects were similar to those observed in the 1–10 Hz frequency range (Fig. 6G), with higher pairwise phase consistency values for the 1–10 Hz frequency range. PPC, pairwise phase consistency. Source data are provided as a Source Data file.

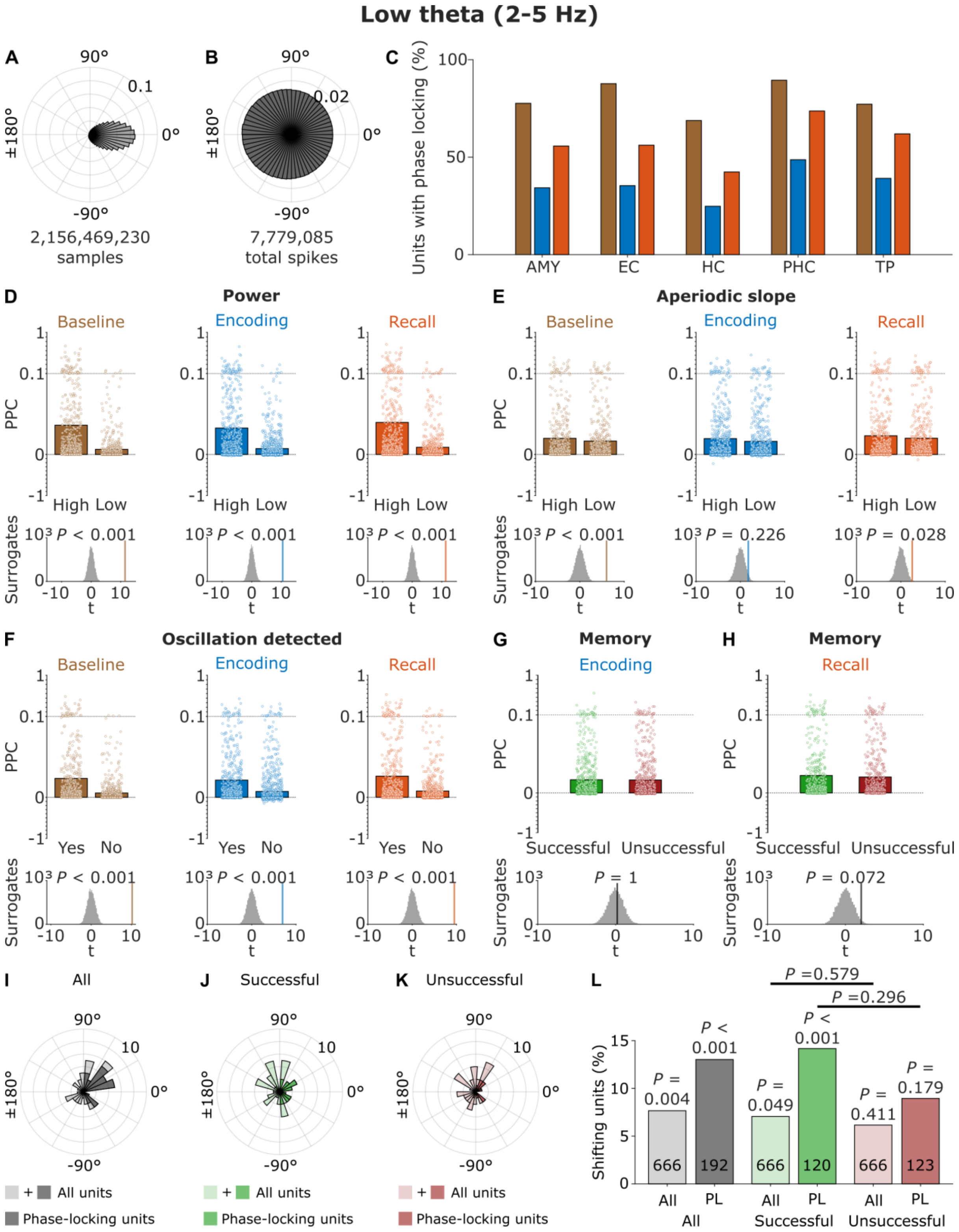

Figure S7. Theta-phase locking to low theta (2–5 Hz). We repeated our main analyses on theta-phase locking for low theta (2–5 Hz), using the filter-Hilbert method for phase estimation. Statistical procedures and numbers of neurons were the same as in the 1–10 Hz analyses. (**A**) Sample-wise phase difference between low-theta phases and generalized phases of the 1–10 Hz filtered signal during cycles with a cycle frequency in the low-theta range. The moderate deviations might be caused by the narrower filtering for low theta compared to the 1–10 Hz signal. (**B**) General phase locking to low theta across the entire experiment. Spikes pooled across all neurons preferentially occurred at the trough. (**C**) Percentages of units with significant phase locking to low theta. (**D–F**) Phase locking to low theta as a function of power, aperiodic slopes, and oscillations. Bars show mean PPC values; dots represent PPC values of individual neurons. Y-axis is expanded between 0 and 0.1 to highlight the range containing most data points. (**G**) Phase locking to low theta as a function of memory performance during encoding.

Bars show mean PPC values; dots represent PPC values of individual neurons. Y-axis is expanded between 0 and 0.1 to highlight the range containing most data points. (**H**) Phase locking to low theta as a function of memory performance during retrieval. Bars show mean PPC values; dots represent PPC values of individual neurons. Y-axis is expanded between 0 and 0.1 to highlight the range containing most data points. (**I–K**) Analysis of theta-phase shifts relative to low theta. Polar histograms show the angular differences between low theta phases during encoding versus retrieval for neurons with significant theta-phase shifts between encoding and retrieval. Results are shown for all units and for units with significant theta-phase locking. (**I**) Results for all segments. (**J**) Results for successful segments. (**K**) Results for unsuccessful segments. (**L**) Percentages of units with significant phase shifts for all (transparent color) and significantly phase locking units (opaque color). Percentages are compared to 5% chance level. All results are Bonferroni corrected for performing each test on low and high theta. PPC, pairwise phase consistency. Source data are provided as a Source Data file.

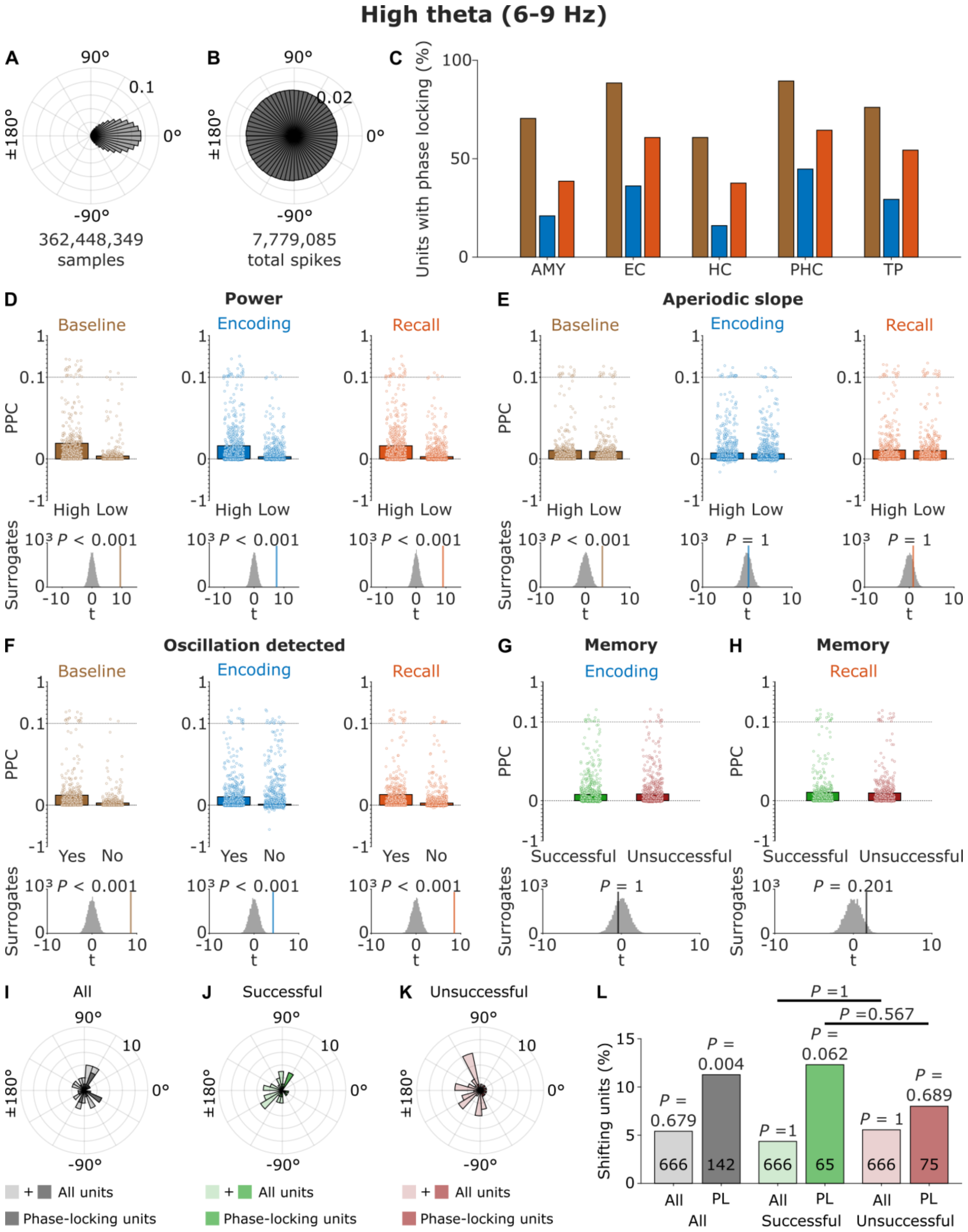

Figure S8. Theta-phase locking to high theta (6–9 Hz). We repeated our main analyses on theta-phase locking for high theta (6–9 Hz), using the filter-Hilbert method for phase estimation. Statistical procedures and numbers of neurons were the same as in the 1–10 Hz analyses. (**A**) Sample-wise phase difference between high-theta phases and generalized phases of the 1–10 Hz filtered signal during cycles with a cycle frequency in the high-theta range. The moderate deviations might be caused by the narrower filtering for high theta compared to the 1–10 Hz signal. (**B**) General phase locking to high theta across the entire experiment. Spikes pooled across all neurons preferentially occurred at the trough. (**C**) Percentages of units with significant phase locking to high theta. (**D–F**) Phase locking to high theta as a function of power, aperiodic slopes, and oscillations. Bars show mean PPC values; dots represent PPC values of individual neurons. Y-axis is expanded between 0 and 0.1 to highlight the range containing most data points. (**G**) Phase locking to high theta as a function of memory performance during encoding. Bars show mean PPC values; dots represent PPC values of individual neurons. Y-axis is expanded between 0 and 0.1 to

highlight the range containing most data points. (**H**) Phase locking to high theta as a function of memory performance during retrieval. Bars show mean PPC values; dots represent PPC values of individual neurons. Y-axis is expanded between 0 and 0.1 to highlight the range containing most data points. (**I–K**) Polar histograms show the angular differences between high theta phases during encoding versus retrieval for neurons with significant theta-phase shifts between encoding and retrieval. Results are shown for all units and for units with significant theta-phase locking. (**I**) Results for all segments. (**J**) Results for successful segments. (**K**) Results for unsuccessful segments. (**L**) Percentages of units with significant phase shifts for all (transparent color) and significantly phase locking units (opaque color). Percentages are compared to 5% chance level. All results are Bonferroni corrected for performing each test on low and high theta. PPC, pairwise phase consistency. Source data are provided as a Source Data file.

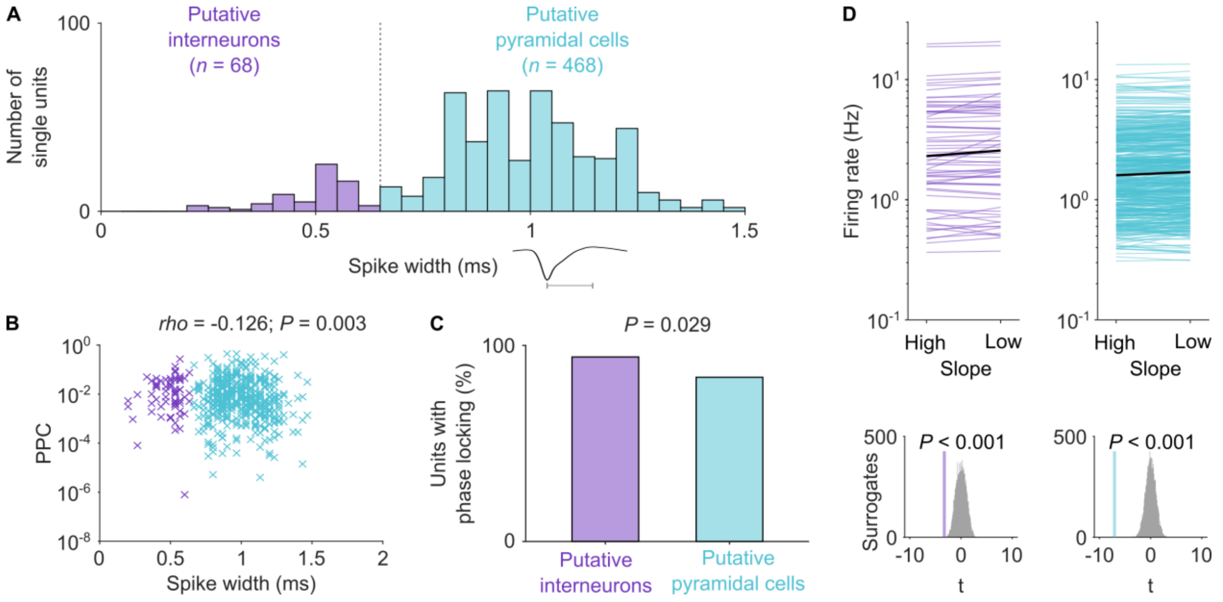

Figure S9. Theta-phase locking of putative interneurons and putative pyramidal cells. (**A**) Distribution of spike width across single units (*n* = 536). Spike width was measured from the minimum to subsequent maximum of the average waveform. We classified single units with a spike width ≤0.65 ms as putative interneurons and >0.65 ms as putative pyramidal cells[2,3]. (**B**) Spike width was negatively correlated with pairwise phase consistency (PPC; Spearman's *rho* = -0.126, *P* = 0.003, *n* = 536 single units). (**C**) The percentage of theta-phase locking units was higher for putative interneurons than putative pyramidal cells (two-sided surrogate analysis with unit-label shuffling, Δ = 10%, *P* = 0.029). (**D**) Firing rate as a function of aperiodic slope (as in Fig. 6B) for putative interneurons (left) and pyramidal cells (right). Each line, one unit. Black lines connect median firing rates. In both cases, firing rates were higher during periods of lower aperiodic slopes. Bottom: Empirical *t*-values of paired *t*-tests comparing firing rates between conditions (vertical lines), along with distributions of surrogate *t*-values (gray histograms). Surrogates were obtained by randomly shuffling the condition labels (same statistical procedure as in Fig. 6B). PPC, pairwise phase consistency. Source data are provided as a Source Data file.

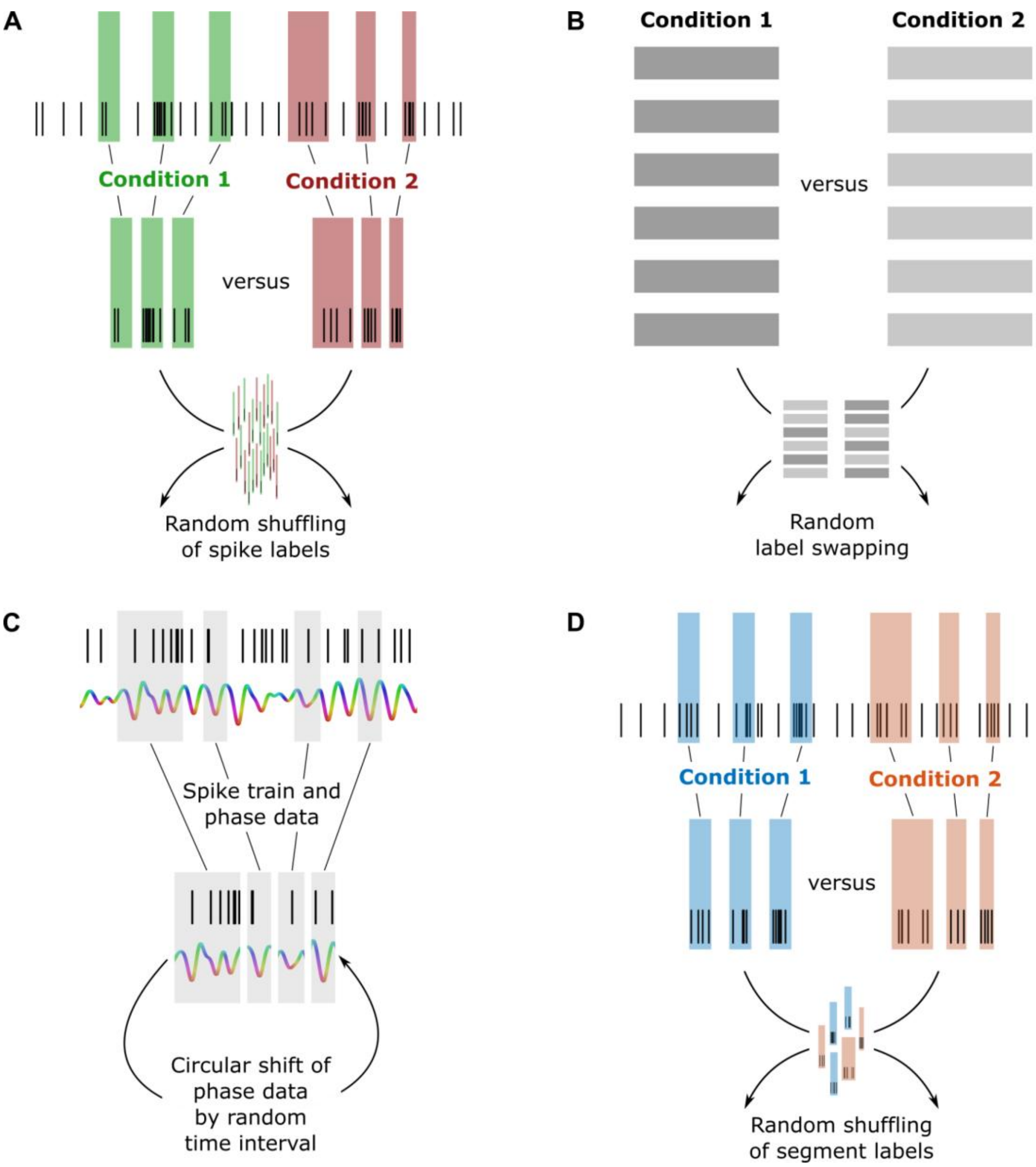

Figure S10. Surrogate tests. (**A**) Permutation procedure for shuffling spike labels across conditions. We randomly rearranged the spike labels to create surrogate datasets. This procedure was used to maintain the original number of spikes for both conditions, for instance when comparing phase-locking strength between two conditions. (**B**) Permutation procedure with label swapping. For each pair, the labels were randomly swapped or maintained to create surrogate data. The original pairing is kept, only the assignment to the conditions is permuted. This procedure was used to compare paired data. (**C**) Permutation procedure with circular shift. Concatenated phase data was circularly shifted by random time intervals relative to the spike train to create surrogate datasets. This procedure was used to preserve the basic structure of the phase data, for instance when testing the significance of a neuron's phase locking. (**D**) Permutation procedure for shuffling segment labels across conditions. We randomly rearranged the segment labels to create surrogate datasets. This procedure was used to maintain the original number and timing of spikes within segments, for instance when comparing preferred theta phases during encoding and retrieval.

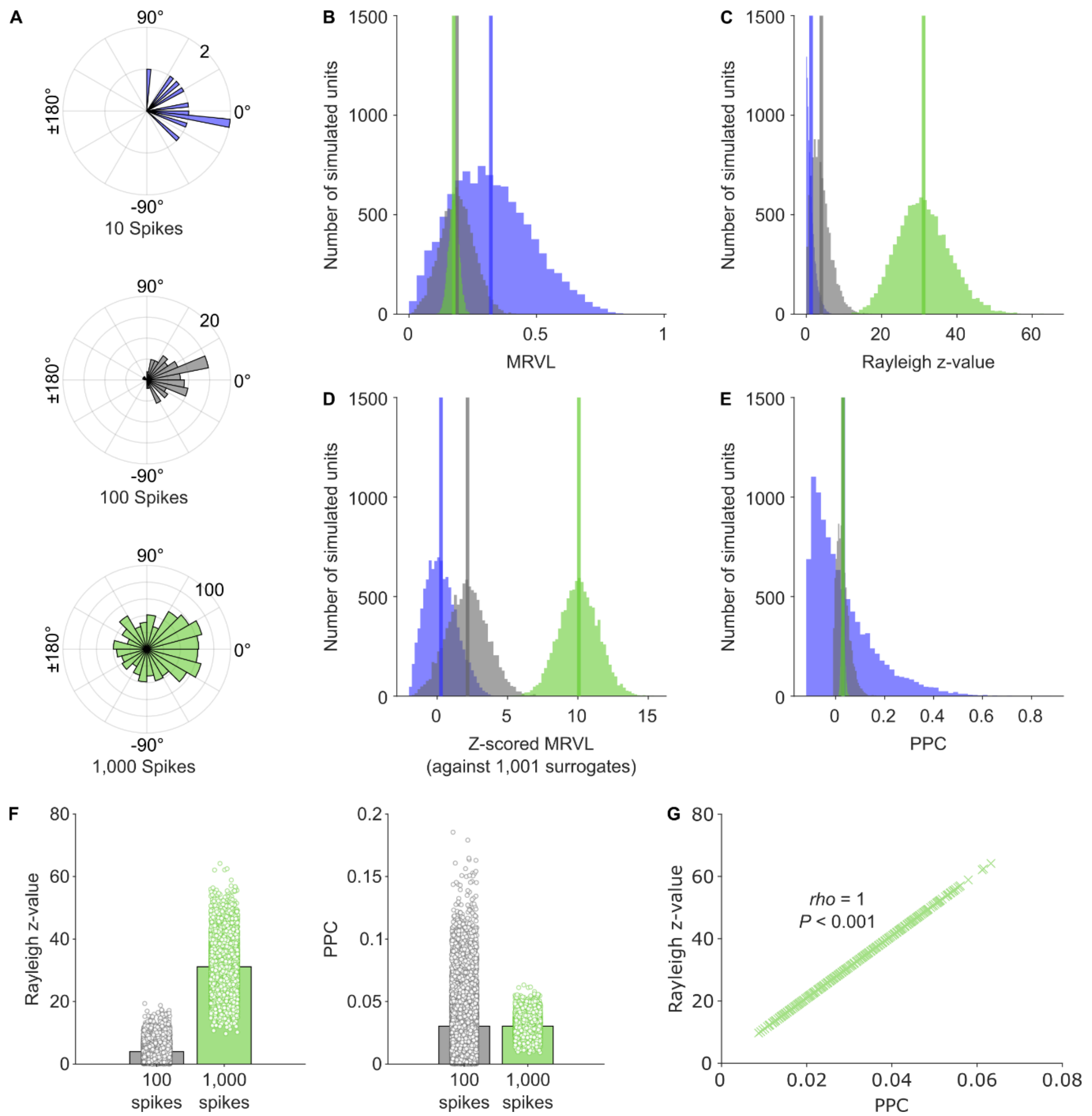

Figure S11. Spike counts influence test statistics for phase-locking strength. To better understand test statistics for assessing phase-locking strength, we simulated 10,000 units with phase locking and applied different test statistics on these simulated data. For each unit, we randomly sampled 250 phase angles from a Von Mises distribution centered around 0°. We added 750 completely random phase angles, resulting in a total of 1,000 phase angles with a preference towards 0°. We considered these simulated phase angles comparable to the phase angles of phase-locking units. (**A**) For each unit, we estimated phase locking-strengths on three different spike counts by separately analyzing 10 (blue), 100 (gray), and all (green) of the 1,000 phase angles. Examples for one unit are shown. (**B**) We calculated the distribution of mean resultant vector lengths (MRVLs) across units separately for the 10-spikes, 100-spikes, and 1000-spikes conditions. Mean MRVLs (vertical lines) decreased with increasing numbers of spikes. (**C**) We calculated the distributions of Rayleigh *z*-values. Mean Rayleigh *z*-values (vertical lines) increased with increasing numbers of spikes. (**D**) We *z*-scored each unit's MRVL against the MRVLs from 1,001 completely random phase angle distributions with a matching spike count. Mean *z*-scored MRVLs (vertical lines) increased with increasing numbers of spikes. (**E**) We calculated the distributions of pairwise phase consistency-values (PPC values). While the variance decreased with increasing numbers of spikes, mean PPC was independent of the spike count. Note the relatively low PPC values close to 0 despite the presence of phase locking. Based on these simulation results, we decided to use the PPC in our analyses because it was not influenced by the number of spikes. (**F**) Histograms of Rayleigh *z*-values (left) and PPC values (right) comparing the 100-spikes and 1000-spikes conditions. Bars indicate means, individual values for each simulated unit are overlaid as dots. While the Rayleigh test did not allow for direct comparisons between conditions with different spike counts, the mean PPC was directly comparable between the two conditions. (**G**) Within the 1000-spikes condition, Rayleigh *z*-values correlated perfectly with the PPC values. The Rayleigh test can thus be used as an alternative to the PPC if conditions with the same spike count are compared (e.g., after subsampling).

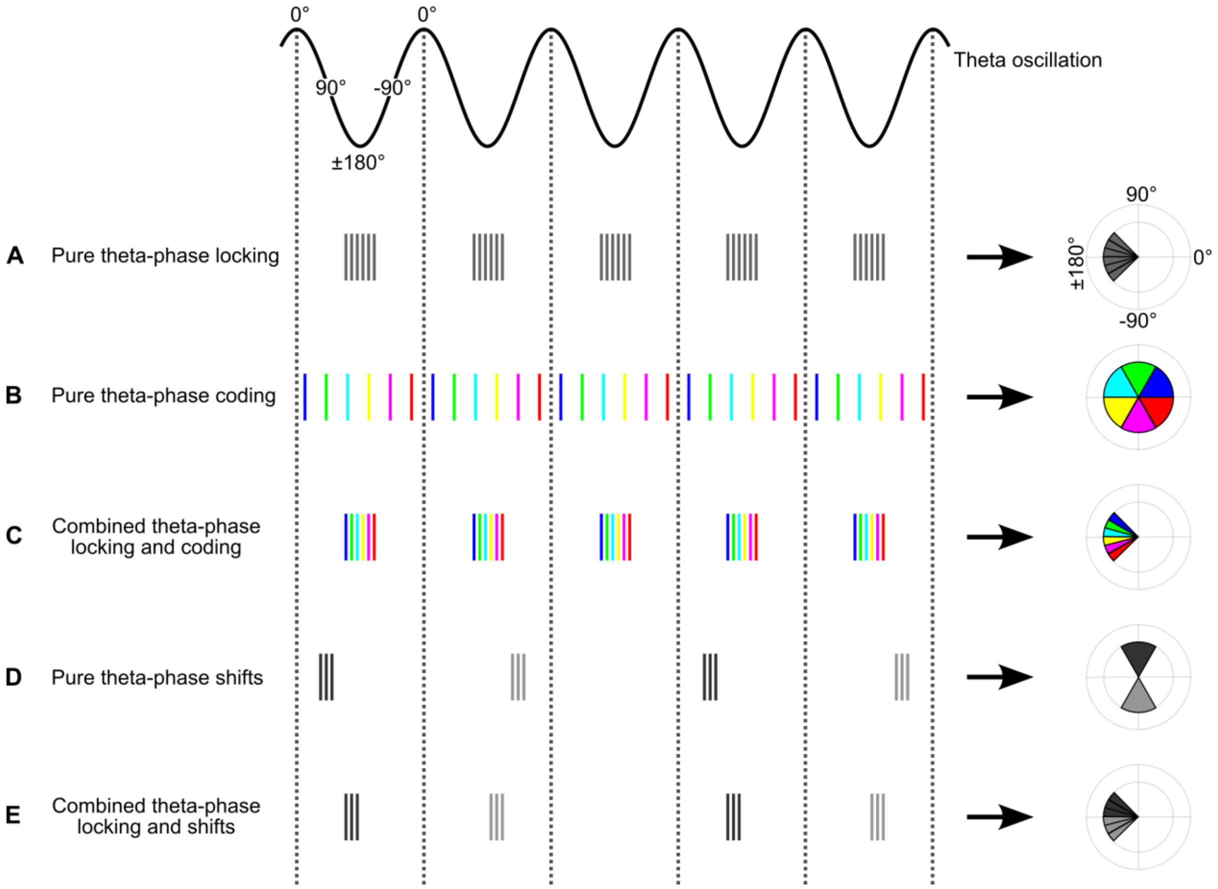

Figure S12. Illustration of the concepts of theta-phase locking, theta-phase coding, and theta-phase shifts. (**A**) Pure theta-phase locking. A neuron activates at similar phases over time, for example at the oscillatory troughs. The theta phases at which the neuron spikes do not differ between different stimuli, and they do not shift between encoding and retrieval. Each vertical line represents an action potential. Schematic polar histogram on the right shows the phase distribution of the action potentials, illustrating the presence of theta-phase locking. (**B**) Pure theta-phase coding in the absence of theta-phase locking. For specific stimuli, the neuron spikes at similar theta phases over time. When pooling across the different stimuli, the neuron spikes at different theta phases because of which there is no overall theta-phase locking. Each vertical line represents an action potential, and different colors indicate different stimuli during which the action potentials occur. Schematic polar histogram on the right shows that different stimuli occupy different angles in phase space. (**C**) Combined theta-phase locking and coding. A neuron generally spikes around the oscillatory trough, leading to theta-phase locking, while also spiking at different theta phases for different stimuli, thus supporting a stimulus-specific phase code. (**D**) Pure theta-phase shifts in the absence of theta-phase locking. The theta phases during encoding are very different from the theta phases during recall. Each vertical line represents an action potential, and the different shades of gray represent encoding and retrieval processes during which the action potentials occur. All theta phases combined, there is no unimodal theta-phase locking, as illustrated in the polar histogram on the right. Strong theta-phase shifts between encoding and retrieval impede stimulus-specific phase coding as the stimulus-specific phase codes would not be stable over time. (**E**) Combined theta-phase locking and shifts. A neuron generally spikes around the oscillatory trough, leading to theta-phase locking, while also shifting its theta phase between encoding and retrieval, thus leading to a separation of these two conditions.

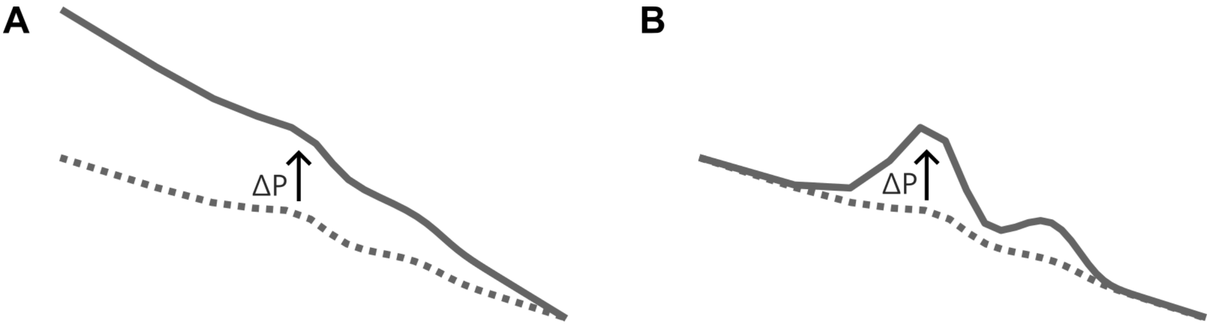

Figure S13. Two different possible reasons for increases in absolute power. (**A**) Absolute power increases (ΔP > 0; black arrow) because of an increased aperiodic slope and an increased y-axis offset, indicated by the upward shift from the dotted reference line to the solid line. There is no increase in narrowband (oscillatory) power. (**B**) Absolute power increases (ΔP > 0) because of increased narrowband (oscillatory) power, indicated by the more pronounced wave above the dotted reference line. Adapted from previous studies[4,5].

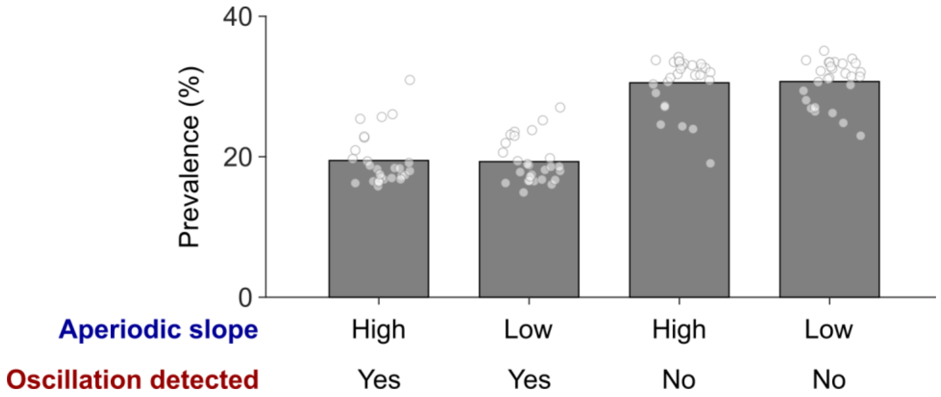

Figure S14. Prevalence of high and low aperiodic slopes in the presence and absence of clear theta oscillations. Bar graph shows the prevalence of high and low aperiodic slopes (median split within each session) during periods with and without clear theta oscillations (bars, means; dots, individual sessions). Theta oscillations were detected in approximately 38% of the signal, with similar proportions (~19%) occurring during high versus low aperiodic slopes (two-sided paired *t*-test, $t(26) = 0.646$, $P = 0.524$; $n = 27$ sessions). Accordingly, the prevalence of high and low aperiodic slopes was also similar during periods without theta oscillations (two-sided paired *t*-test, $t(26) = -0.693$, $P = 0.494$; $n = 27$ sessions). Source data are provided as a Source Data file.

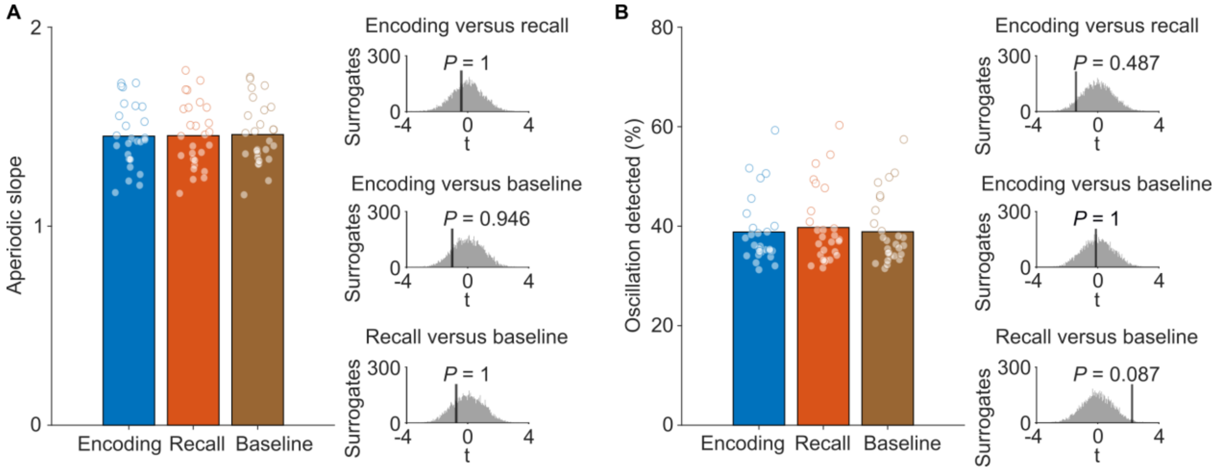

Figure S15. Aperiodic slope and oscillation presence during encoding, recall, and baseline. Analysis of the steepness of aperiodic slopes and the percentage of theta oscillations during different task periods. Recall and baseline periods are subsampled to match the durations of the encoding periods. (**A**) Steepness of the aperiodic slope during encoding, recall, and baseline (left). There were no significant differences in slope steepness between the three conditions (right; two-sided surrogate analysis with session-label swapping: encoding versus recall, $t(26) = -0.419$, $P_{corr.} = 1$; encoding versus baseline, $t(26) = -1.014$, $P_{corr.} = 0.946$; recall versus baseline, $t(26) = -0.752$, $P_{corr.} = 1$; $n = 27$ sessions; Bonferroni corrected for three tests). Bars show mean values; dots represent values of individual sessions. (**B**) Percentage of detected oscillations during encoding, recall, and baseline (left). There were no significant differences between the three conditions (right; two-sided surrogate analysis with session-label swapping: encoding versus recall, $t(26) = -1.422$, $P_{corr.} = 0.487$; encoding versus baseline, $t(26) = -0.121$, $P_{corr.} = 1$; recall versus baseline, $t(26) = 2.248$, $P_{corr.} = 0.087$; $n = 27$ sessions; Bonferroni corrected for three tests). Bars show mean values; dots represent values of individual sessions. Source data are provided as a Source Data file.

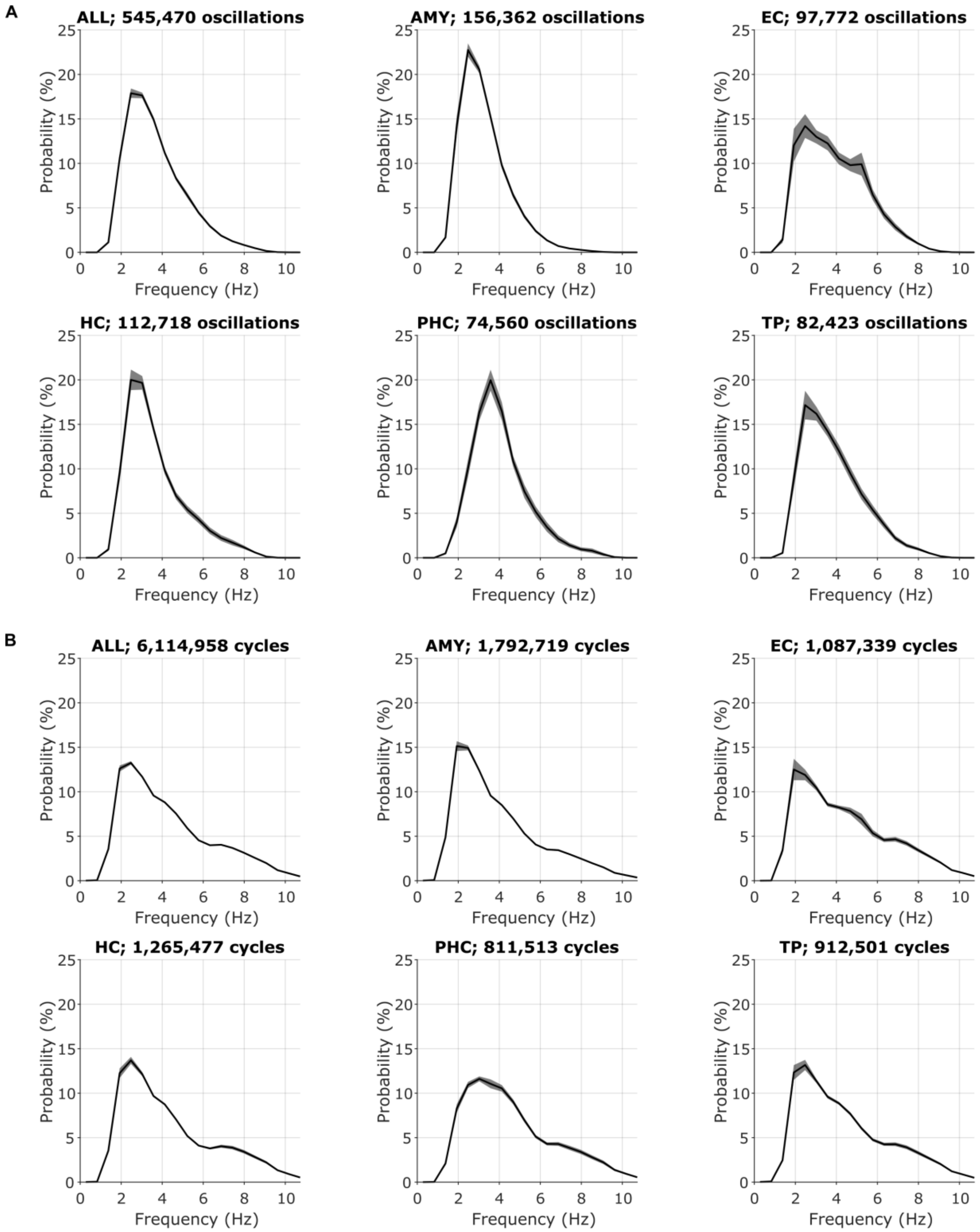

Figure S16. Distributions of theta frequencies for different brain regions. (**A**) Plots show probability distributions of frequencies of detected 1–10 Hz oscillations across microwires, identified using Bycycle[6]. Gray shaded areas, standard error of the mean across microwires. (**B**) Plots show probability distributions of frequencies of detected 1–10 Hz cycles identified by the generalized phase approach across microwires. Gray shaded areas, standard error of the mean across microwires. Note that not all these cycles are part of a detected oscillation. ALL, all brain regions; AMY, amygdala; EC, entorhinal cortex; HC, hippocampus; PHC, parahippocampal cortex; TP, temporal pole. Source data are provided as a Source Data file.

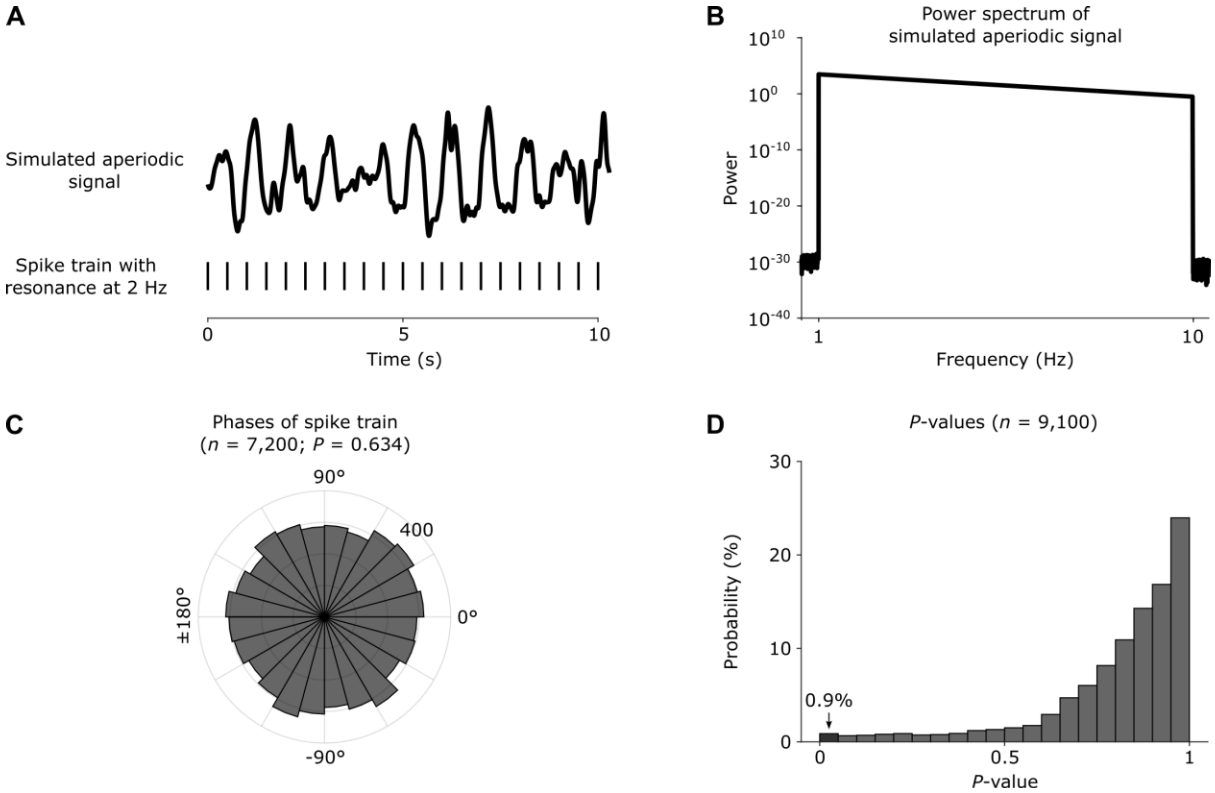

Figure S17. Theta resonance of individual neurons in combination with a purely aperiodic local field potential does not explain theta-phase locking. We simulated one hundred aperiodic signals between 1–10 Hz with a duration of 3,600 seconds. For each of these signals, we simulated 91 spike trains with theta resonance at linearly increasing frequencies from 1 to 10 Hz. (**A**) Ten seconds of an example aperiodic signal and a spike train with resonance at 2 Hz. (**B**) The power spectrum of the example signal in log-log space confirms that it contains 1/f-like aperiodic activity between 1–10 Hz but no oscillatory peaks. We simulated the slope of the aperiodic activity with a steepness of 2. (**C**) Polar histogram showing the distribution of spike-associated phases of the example spike train and signal. We computed the pairwise phase consistency (PPC = $-8.412 * 10^{-5}$) and compared it to 101 surrogate PPC values obtained from random phase distributions. The rank of the empirical PPC in the surrogate PPC distribution was subtracted from one to compute a *P*-value of 0.634, indicating a uniform distribution of phases. Accordingly, there is no theta-phase locking in this simulation despite the spike train's perfect theta resonance at 2 Hz. **(D)** Histogram showing the distribution of *P*-values (obtained using the same procedure as in **C**) for all simulated aperiodic signals and associated spike trains. Only 0.9% of the spike trains had a *P*-value < 0.05, indicating a negligible influence of theta resonance of individual neurons on theta-phase locking. Hence, our findings on residual theta-phase locking in the absence of clear oscillations is most likely explained by subthreshold oscillatory activity that was missed by our method of oscillation detection.

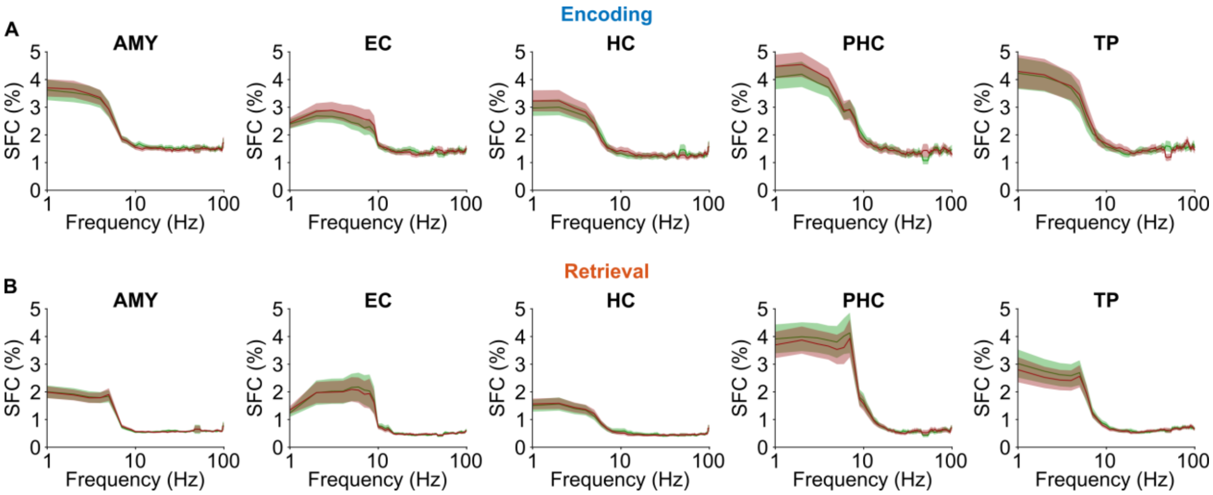

Figure S18. Spike-field coherence (SFC) as a function of memory performance in different medial temporal lobe regions. (**A**) Spike-field coherence during encoding. (**B**) Spike-field coherence during retrieval. Shaded areas, mean ± standard error of the mean across units. Green, successful trials; red, unsuccessful trials. AMY, amygdala; EC, entorhinal cortex; HC, hippocampus; PHC, parahippocampal cortex; TP, temporal pole. Source data are provided as a Source Data file.

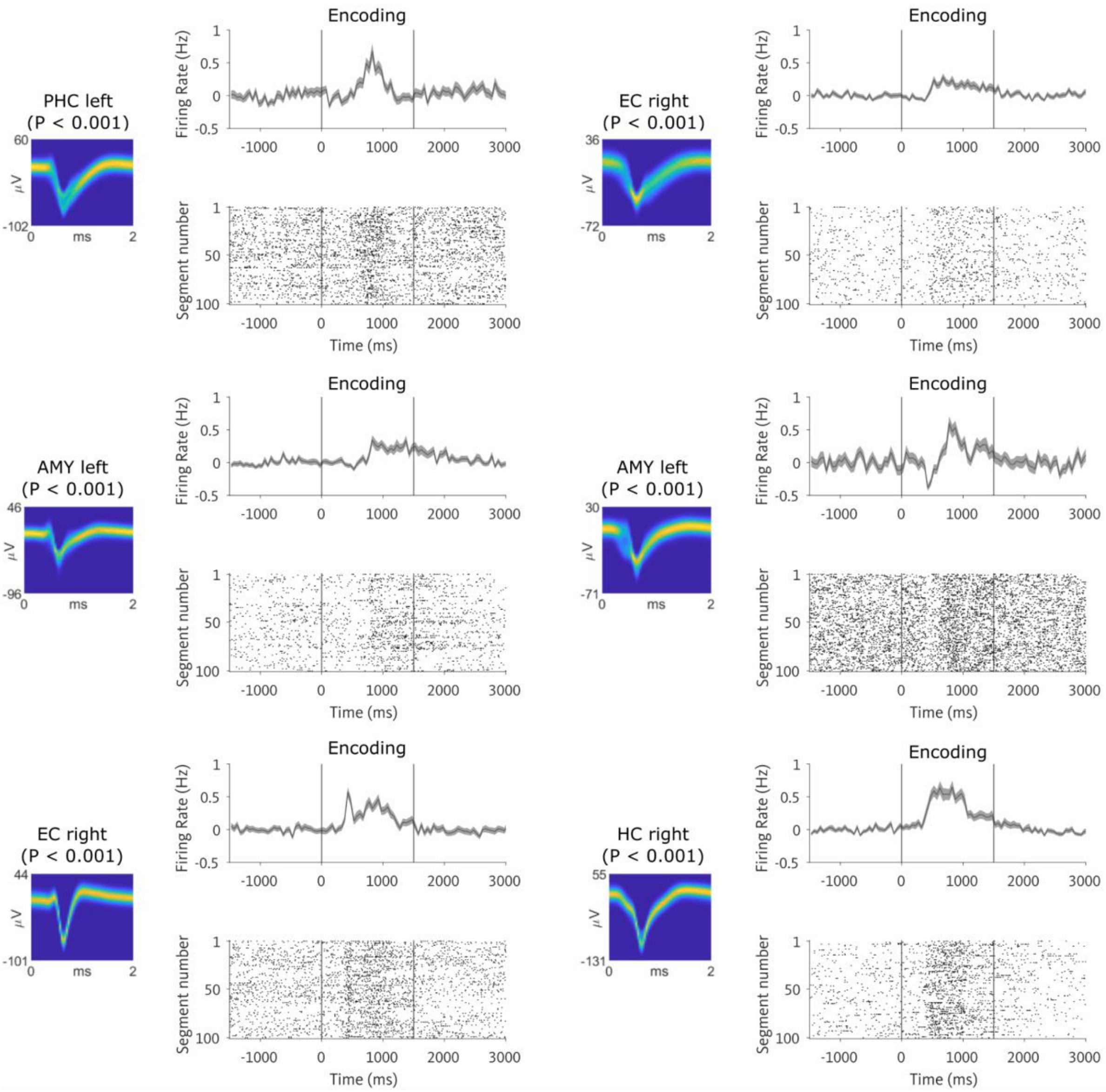

Figure S19. Examples of object-responsive units. We identified object-responsive units as those units that exhibited a significant increase in firing during the encoding period of the task (in comparison to baseline periods). This figure shows six example object-responsive units. For each unit, the top plot shows the baseline-corrected mean firing rate across encoding segments. Gray shaded area indicates the standard error of the mean. Vertical lines indicate the start and end time of the encoding period. Bottom raster plots show spiking activity during each encoding segment. Spike waveforms are shown as density plots on the left. AMY, amygdala; EC, entorhinal cortex; HC, hippocampus; PHC, parahippocampal cortex. Source data are provided as a Source Data file.

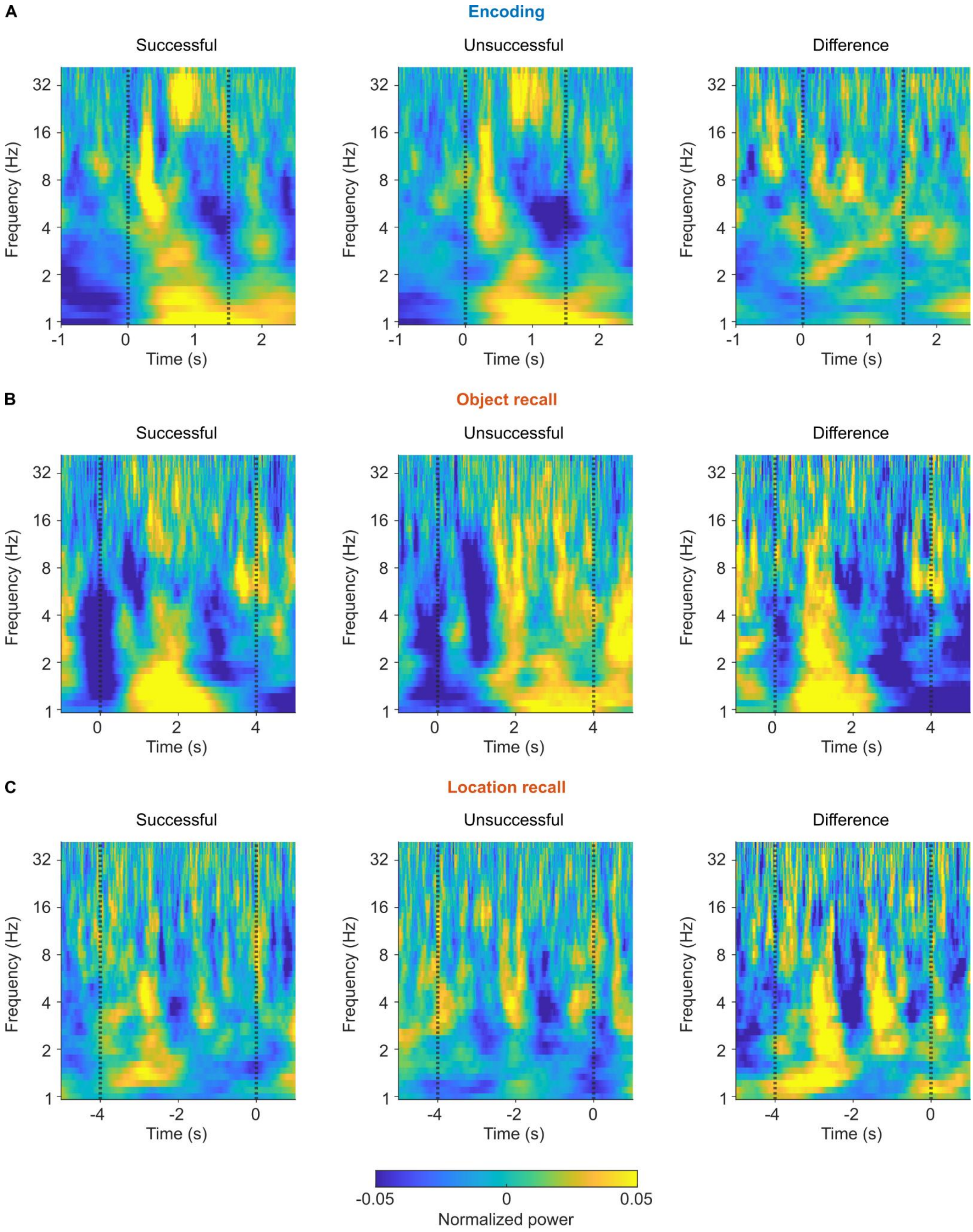

Figure S20. Time–frequency-resolved power during encoding and retrieval. Plots show the grand mean normalized power in the frequency range of 1 to 40 Hz during successful, unsuccessful, and successful versus unsuccessful encoding (**A**), object recall (**B**), and location recall (**C**). Dotted vertical lines indicate the start and end time of the segments. For encoding and object recall, time = 0 s indicates the start of the segment. For location recall, time = 0 s is referenced to the segment end. We included power spectrograms of all microwires with at least one unit in the analysis. We computed spectral power in time bins of 10 ms during encoding segments (1.5 s duration), object retrieval segments (4 s duration), and the last 4 s of the location retrieval segments including 5-s buffer periods before and after to avoid edge artifacts. To obtain the power spectrogram of each microwire, we calculated a continuous Morlet wavelet transform (number of cycles, 5) at 40 logarithmically spaced frequencies between 1 and 40 Hz. The resulting power values of each microwire were log-transformed and then normalized separately for each frequency and separately for encoding, object recall, and location recall (including the segments and their buffer periods).

For each session, we calculated the mean power spectrum across all segments and then across all microwires with at least one unit (from various brain regions). In a last step, we calculated the grand mean across all sessions. We did not observe significant power differences between successful and unsuccessful segments (two-sided cluster-based permutation tests, 1001 permutations: encoding (0–1.5 s), largest positive cluster, $t(26) = 259.204$, $P = 0.185$, largest negative cluster, $t(26) = -59.745$, $P = 0.635$; object recall (0–4 s), largest positive cluster, $t(26) = 1295.597$, $P = 0.058$, largest negative cluster, $t(26) = -474.827$, $P = 0.253$; location recall (-4–0 s), largest positive cluster, $t(26) = 352.748$, $P = 0.285$, largest negative cluster, $t(26) = -283.507$, $P = 0.363$; $n = 27$ sessions). Source data are provided as a Source Data file.

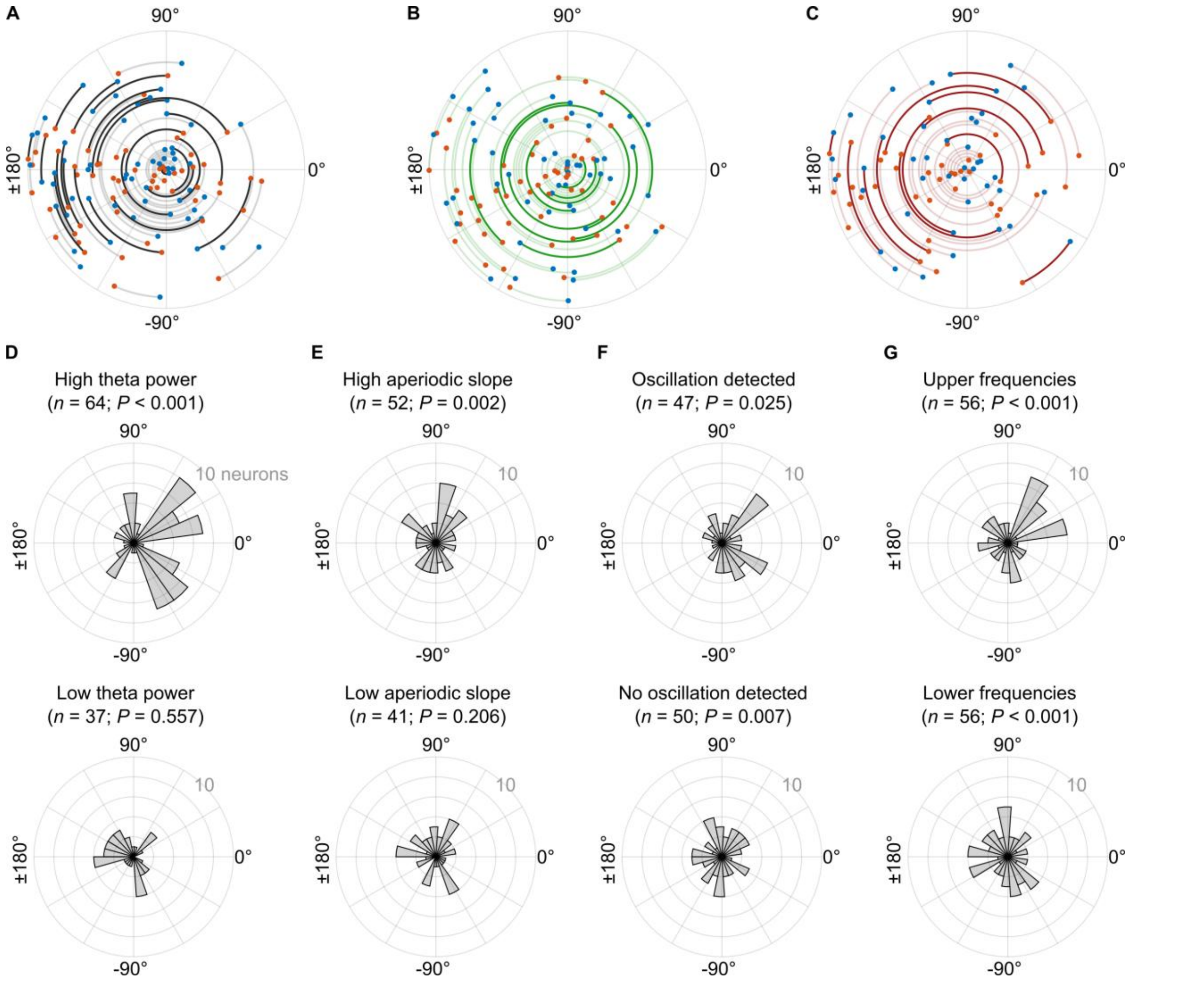

Figure S21. Encoding–retrieval phase shifts as a function of memory performance, theta power, aperiodic slope, clear theta oscillations, and theta frequency. (**A–C**) Polar plots show the angular shifts between theta phases during encoding (blue dots) versus retrieval (orange dots) for neurons with significant theta-phase shifts between encoding and retrieval. Results are shown for all units (all lines) and for units with significant theta-phase locking (darker lines). Units are sorted from center to periphery by decreasing phase difference (larger phase differences toward the center). (**A**) Results for all segments (n = 62 significant units from the pool of all units; n = 26 significant units from the pool of significantly phase-locking units). (**B**) Results for successful segments (n = 54 and n = 14, respectively). (**C**) Results for unsuccessful segments (n = 43 and n = 12, respectively). (**D–G**) Polar histograms show angular theta-phase differences of neurons with significant theta-phase shifts between encoding and retrieval as a function of theta power, aperiodic slope, theta oscillations, and frequency. A positive angular difference corresponds to a shift from an earlier phase during encoding to a later phase during retrieval. For different conditions, the number of phase-shifting units and the P-values of a one-sided binomial test are displayed. Results are Bonferroni corrected for two tests per condition. (**D**) Phase shifts for high and low theta power. (**E**) Phase shifts for high and low aperiodic slope. (**F**) Phase shifts in the presence and absence of theta oscillations. (**G**) Phase shifts for upper and lower spike-associated instantaneous frequencies (unit-wise median split). To test for a preferred angle in upper-frequency phase shifts, we computed the pairwise phase consistency (PPC = 0.005) and compared it against 10,001 surrogate PPC values from random distributions. The resulting P-value of 0.277 indicated no significant angular preference. Source data are provided as a Source Data file.

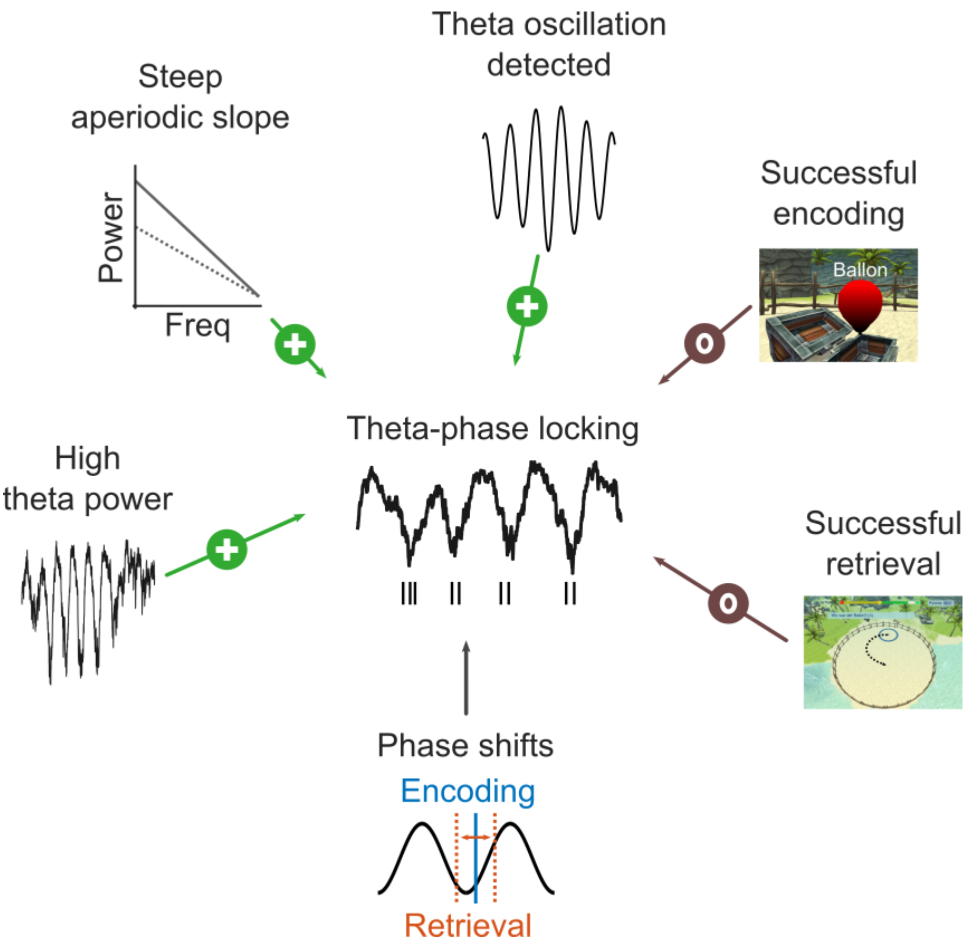

Figure S22. Summary schematic. Neuronal theta-phase locking increased during periods of elevated theta power, when aperiodic activity exhibited steeper slopes, and when clear theta oscillations were detected. Theta-phase locking was similarly strong during the successful and unsuccessful encoding and retrieval of memories. Some neurons changed their preferred theta phases between encoding and retrieval. The virtual beach environment (screenshots to the right) was created using the Unity 3D graphics engine and the Hand-painted Island pack (obtained from the Unity asset store under the Standard Unity Asset Store EULA; https://assetstore.unity.com/packages/3d/environments/fantasy/hand-painted-island-pack-36959#asset_quality).

References

1. Davis, Z. W., Muller, L., Martinez-Trujillo, J., Sejnowski, T. & Reynolds, J. H. Spontaneous travelling cortical waves gate perception in behaving primates. *Nature* **587**, 432–436 (2020).
2. Gast, H. *et al.* Burst firing of single neurons in the human medial temporal lobe changes before epileptic seizures. *Clinical Neurophysiology* **127**, 3329–3334 (2016).
3. Ison, M. J. *et al.* Selectivity of pyramidal cells and interneurons in the human medial temporal lobe. *Journal of Neurophysiology* **106**, 1713–1721 (2011).
4. Herweg, N. A., Solomon, E. A. & Kahana, M. J. Theta Oscillations in Human Memory. *Trends in Cognitive Sciences* **24**, 208–227 (2020).
5. Donoghue, T. *et al.* Parameterizing neural power spectra into periodic and aperiodic components. *Nat Neurosci* **23**, 1655–1665 (2020).
6. Cole, S. & Voytek, B. Cycle-by-cycle analysis of neural oscillations. *Journal of Neurophysiology* **122**, 849–861 (2019).
