# Supplementary material for: Theta-phase locking of single neurons during human spatial memory
Source: Nat Commun. 2025 Aug 11;16:7402. doi: 10.1038/s41467-025-62553-9 (PMC12339722; doi:10.1038/s41467-025-62553-9)
Supplement: Supplementary file 2 — Reporting Summary [file 41467_2025_62553_MOESM2_ESM.pdf]

nature portfolio

Corresponding author(s): Tim Guth

Last updated by author(s): 2025/06/03

Reporting Summary

Nature Portfolio wishes to improve the reproducibility of the work that we publish. This form provides structure for consistency and transparency in reporting. For further information on Nature Portfolio policies, see our Editorial Policies and the Editorial Policy Checklist.

Statistics

For all statistical analyses, confirm that the following items are present in the figure legend, table legend, main text, or Methods section.

n/a	Confirmed	
☐	☒	The exact sample size (n) for each experimental group/condition, given as a discrete number and unit of measurement
☐	☒	A statement on whether measurements were taken from distinct samples or whether the same sample was measured repeatedly
☐	☒	The statistical test(s) used AND whether they are one- or two-sided<br>*Only common tests should be described solely by name; describe more complex techniques in the Methods section.*
☐	☒	A description of all covariates tested
☐	☒	A description of any assumptions or corrections, such as tests of normality and adjustment for multiple comparisons
☐	☒	A full description of the statistical parameters including central tendency (e.g. means) or other basic estimates (e.g. regression coefficient) AND variation (e.g. standard deviation) or associated estimates of uncertainty (e.g. confidence intervals)
☐	☒	For null hypothesis testing, the test statistic (e.g. F, t, r) with confidence intervals, effect sizes, degrees of freedom and P value noted<br>*Give P values as exact values whenever suitable.*
☒	☐	For Bayesian analysis, information on the choice of priors and Markov chain Monte Carlo settings
☐	☒	For hierarchical and complex designs, identification of the appropriate level for tests and full reporting of outcomes
☐	☒	Estimates of effect sizes (e.g. Cohen's d, Pearson's r), indicating how they were calculated

Our web collection on statistics for biologists contains articles on many of the points above.

Software and code

Policy information about availability of computer code

Data collection	The spatial memory task was developed with Unity 3D (Unity Technologies, San Francisco, CA, USA). Neurophysiological data were collected using a NeuroPort System (Blackrock Microsystems, Salt Lake City, UT, USA) and hybrid depth electrodes (Ad-Tech, Oak Creek, WI, USA). See the Methods section for a detailed description.
Data analysis	Data analyses were carried out in MATLAB 2021a and 2023a (The MathWorks, Inc., Natick, MA, USA). Custom Matlab code is available at https://github.com/BonnSpatialMemoryLab/GuthPhaseLocking2025. Circular statistics were performed using the CircStat toolbox, version 1.21.0.0 (Berens, 2009). Local field potentials were analyzed using FieldTrip, version 20210912 (Oostenveld et al., 2011). Spike sorting was performed using Wave_Clus 3 (Chaure et al., 2018).

For manuscripts utilizing custom algorithms or software that are central to the research but not yet described in published literature, software must be made available to editors and reviewers. We strongly encourage code deposition in a community repository (e.g. GitHub). See the Nature Portfolio guidelines for submitting code & software for further information.

Data

Policy information about availability of data

All manuscripts must include a data availability statement. This statement should provide the following information, where applicable:

- Accession codes, unique identifiers, or web links for publicly available datasets
- A description of any restrictions on data availability
- For clinical datasets or third party data, please ensure that the statement adheres to our policy

Data to produce all figures are provided at https://github.com/BonnSpatialMemoryLab/GuthPhaseLocking2025 withing the directory "SourceData_20250129". Data analyses were performed with custom Matlab code and using the Matlab toolboxes CircStat (Berens, 2009) and FieldTrip version 20210912 (Oostenveld et al., 2011). All custom Matlab code generated during this study for data analysis is available at https://github.com/BonnSpatialMemoryLab/GuthPhaseLocking2025. Source data are provided with this paper.

Research involving human participants, their data, or biological material

Policy information about studies with human participants or human data. See also policy information about sex, gender (identity/presentation), and sexual orientation and race, ethnicity and racism.

Reporting on sex and gender	Of the 18 participants, 10 were female and 8 male. The classification of sex/gender was based on the self-reported information provided by the participants. No sex- or gender-specific analyses were performed in this study because of low sample sizes and because no sex- or gender-specific differences were expected.
Reporting on race, ethnicity, or other socially relevant groupings	Information on race, ethnicity, or other socially relevant groupings was not assessed. No analyses specific to race, ethnicity, or other socially relevant groupings were performed in this study.
Population characteristics	We tested 18 human participants (10 female; age range, 19–61 years; mean age ± SEM, 38 ± 3 years) who were epilepsy patients undergoing treatment for pharmacologically intractable epilepsy at the Freiburg Epilepsy Center, Freiburg im Breisgau, Germany. See Supplementary Table S1 for additional participant information.
Recruitment	Epilepsy patients who were undergoing invasive electrophysiological recordings for clinical purposes were recruited if they were able and willing to perform the task.
Ethics oversight	The study conformed to the guidelines of the ethics committee of the University Hospital Freiburg, Freiburg im Breisgau, Germany ("Ethik-Kommission der Albert-Ludwigs-Universität Freiburg") and written informed consent was obtained from all participants.

Note that full information on the approval of the study protocol must also be provided in the manuscript.

Field-specific reporting

Please select the one below that is the best fit for your research. If you are not sure, read the appropriate sections before making your selection.

☒ Life sciences ☐ Behavioural & social sciences ☐ Ecological, evolutionary & environmental sciences

For a reference copy of the document with all sections, see nature.com/documents/nr-reporting-summary-flat.pdf

Life sciences study design

All studies must disclose on these points even when the disclosure is negative.

Sample size	We analyzed 666 neurons from 18 participants who contributed a total of 27 sessions. No a-priori sample-size calculation was performed. Sample sizes were determined based on typical sample sizes in the field (Kunz et al., Nature Neuroscience, 2024; Qasim et al., Nature Neuroscience, 2019; Jacobs et al., Nature Neuroscience 2013; Rutishauser et al., Nature, 2010).
Data exclusions	No data were excluded.
Replication	All analyses were performed on the entire available data and significant effects replicated across the underlying samples. No separate replication study was performed.
Randomization	All participants were in the same experimental group. No randomization was required.
Blinding	Subjects were not aware of the goals of the study. There was no subjective measurement or decision that the investigator needed to make during the experiment. All data were analyzed off-line. Data collection and analyses were not performed blind to the conditions of the experiments as conditional information was required for further analyses.

Reporting for specific materials, systems and methods

We require information from authors about some types of materials, experimental systems and methods used in many studies. Here, indicate whether each material, system or method listed is relevant to your study. If you are not sure if a list item applies to your research, read the appropriate section before selecting a response.

Materials & experimental systems

n/a	Involved in the study
☒	☐ Antibodies
☒	☐ Eukaryotic cell lines
☒	☐ Palaeontology and archaeology
☒	☐ Animals and other organisms
☒	☐ Clinical data
☒	☐ Dual use research of concern
☒	☐ Plants

Methods

n/a	Involved in the study
☒	☐ ChIP-seq
☒	☐ Flow cytometry
☐	☒ MRI-based neuroimaging

Plants

Seed stocks: *Report on the source of all seed stocks or other plant material used. If applicable, state the seed stock centre and catalogue number. If plant specimens were collected from the field, describe the collection location, date and sampling procedures.*

Novel plant genotypes: *Describe the methods by which all novel plant genotypes were produced. This includes those generated by transgenic approaches, gene editing, chemical/radiation-based mutagenesis and hybridization. For transgenic lines, describe the transformation method, the number of independent lines analyzed and the generation upon which experiments were performed. For gene-edited lines, describe the editor used, the endogenous sequence targeted for editing, the targeting guide RNA sequence (if applicable) and how the editor was applied.*

Authentication: *Describe any authentication procedures for each seed stock used or novel genotype generated. Describe any experiments used to assess the effect of a mutation and, where applicable, how potential secondary effects (e.g. second site T-DNA insertions, mosiacism, off-target gene editing) were examined.*

Magnetic resonance imaging

Experimental design

Design type: MRI scans were obtained solely for clinical reasons to verify electrode placement and were not part of the experiment.

Design specifications: MRI scans were obtained solely for clinical reasons to verify electrode placement and were not part of the experiment.

Behavioral performance measures: MRI scans were obtained solely for clinical reasons to verify electrode placement and were not part of the experiment.

Acquisition

Imaging type(s): Structural MRI.

Field strength: 3T before electrode implantation; 1.5T after electrode implantation.

Sequence & imaging parameters: Pre-implant 3D T1-weighted MPRAGE (Siemens Prisma, Germany): TR 2,000 ms; TE 2.26 ms; flip angle 12; 1 mm isotropic resolution; 256 x 256 x 160 matrix.
Post-implant 3D T1-weighted MPRAGE (Siemens Avanto, Germany): TR 1,300 ms; TE 2.33 ms; flip angle 15; 0.5 x 0.5 x 1 mm resolution; 512 x 512 x 176 matrix.

Area of acquisition: Whole brain.

Diffusion MRI: ☐ Used ☒ Not used

Preprocessing

Preprocessing software: SPM (https://www.fil.ion.ucl.ac.uk/spm/).

Normalization: Normalization was performed using SPM.

Normalization template: Normalization was performed using the SPM template.

Noise and artifact removal | No noise or artifact removal was used.

Volume censoring | No volume censoring was used.

Statistical modeling & inference

Model type and settings | No statistical modeling was used as MRI scans were only obtained for clinical purposes to verify electrode placement.

Effect(s) tested | No effects were tested as MRI scans were only obtained for clinical purposes to verify electrode placement.

Specify type of analysis: ☒ Whole brain ☐ ROI-based ☐ Both

Statistic type for inference
(See Eklund et al. 2016) | No statistical analyses were performed.

Correction | No statistical analyses were performed and no correction was applied.

Models & analysis

n/a	Involved in the study
☒	☐ Functional and/or effective connectivity
☒	☐ Graph analysis
☒	☐ Multivariate modeling or predictive analysis
